# Hallmarks of DNA replication stress responses in *Escherichia coli* and *Bacillus subtilis*

**DOI:** 10.1093/femsre/fuaf041

**Published:** 2025-08-28

**Authors:** Rubén Torres, Begoña Carrasco, Silvia Ayora, Juan C Alonso

**Affiliations:** Department of Microbial Biotechnology, Centro Nacional de Biotecnología, CNB-CSIC, 3 Darwin St, 28049 Madrid, Spain; Department of Microbial Biotechnology, Centro Nacional de Biotecnología, CNB-CSIC, 3 Darwin St, 28049 Madrid, Spain; Department of Microbial Biotechnology, Centro Nacional de Biotecnología, CNB-CSIC, 3 Darwin St, 28049 Madrid, Spain; Department of Microbial Biotechnology, Centro Nacional de Biotecnología, CNB-CSIC, 3 Darwin St, 28049 Madrid, Spain

**Keywords:** DNA damage tolerance, lesion skipping, fork reversal, template switching, translesion synthesis, R-loop, replication–transcription conflicts

## Abstract

*Escherichia coli* and *Bacillus subtilis* provide well-studied models for understanding how bacteria manage DNA replication stress (RS). These bacteria employ various strategies to detect and stabilize stalled replication forks (RFs), circumvent or bypass lesions, resolve replication–transcription conflicts (RTCs), and resume replication. While central features of responses to RS are broadly conserved, distinct mechanisms have evolved to adapt to their complex environments. In this review, we compare the RS sensors, regulators, and molecular players of these two phylogenetically distant bacteria. The differing roles of the RecA recombinase are used as the touchstone of the distinct strategies each bacterium employs to overcome RS, provided that the fork does not collapse. In *E. coli*, RecA mainly assembles at locations distal from replisomes, promotes global responses, and contributes to circumvent or bypass lesions. RecA assembles less frequently at stalled RFs, and its role in lesion skipping, fork remodeling, RTC resolution, and replication restart remains poorly defined. In contrast, in *B. subtilis*, RecA assembles at stalled forks, fine-tunes damage signaling, and, in concert with RecA-interacting proteins, may facilitate fork remodeling or lesion bypass, overcome RTCs, and contribute to replication restart.

## Teaser

Comparison of *Escherichia coli* and *Bacillus subtilis* replication stress responses.

Abbreviations ATP: Adenosine triphosphate
BER: Base excision repair
CD: Codirectional
c-di-AMP: Cyclic 3′, 5′-diadenosine monophosphate
DDT: DNA damage tolerance
DNAP: DNA polymerase
DSB: Double-strand break
dsDNA: Double-stranded DNA
HE: Holoenzyme
HJ: Holliday junction
HO: Head-on
HPUra: 6-(p-Hydroxyphenylazo)-uracil
LB: Luria-Bertani
MMS: Methyl methanesulfonate
4NQO: 4-nitroquinoline-1-oxide
NER: Nucleotide excision repair
NHEJ: Non-homologous end joining
RER: Ribonucleotide excision repair
RF: Replication fork
R-loop: RNA–DNA hybrid and a displaced ssDNA strand
RNAP: RNA polymerase
rNMP: Ribonucleoside monophosphate
rNTP: Ribonucleotide triphosphate
RS: Replication stress
RTC: Replication–transcription conflict
ssDNA: Single-stranded DNA
SID: Specific interacting domain
TLS: Translesion synthesis
UV: Ultraviolet light
wt: Wild-type

## Introduction

In all living organisms, accurate, efficient, and complete genome duplication is essential for transmitting intact genetic information to the next generation. When the DNA replication complex (replisome) encounters endogenous and/or exogenous threats, it can stall, triggering both local and global DNA damage responses to preserve genome integrity and stability—a phenomenon collectively termed replication stress (RS). In these situations, DNA damage tolerance (DDT) subpathways are crucial, as they help prevent replication fork (RF) collapse, which could otherwise result in deleterious double-strand breaks (DSBs) (reviewed in Mirkin and Mirkin 2005, Ciccia and Elledge 2010, Zeman and Cimprich 2014, Gaillard and Aguilera 2016, Lang and Merrikh 2018).

**Figure 1.**
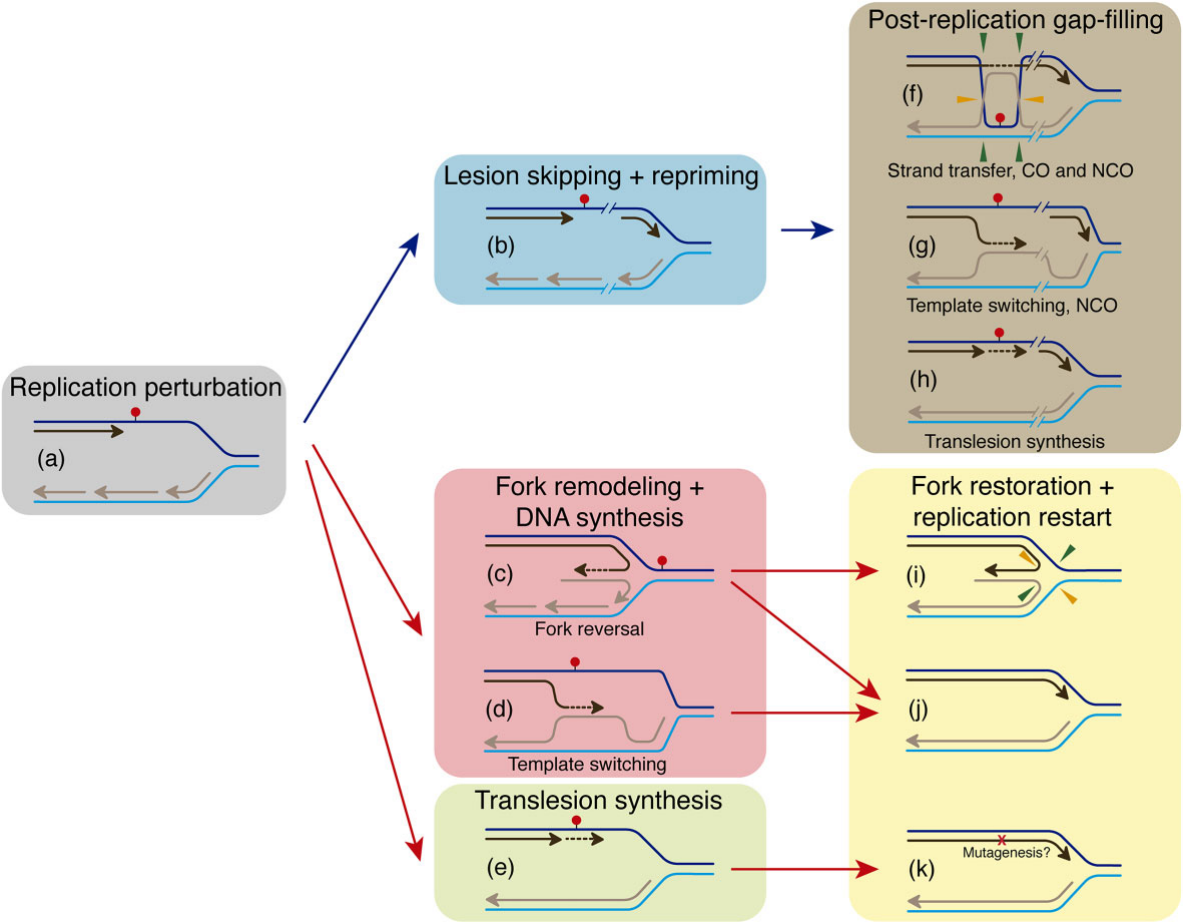
Mechanisms of stalled RF rescue in response to DNA damage. (a) A replicative DNAP stalls upon encountering a lesion (red circle) in the leading-strand template. (b) In the lesion skipping model, leading-strand DNA synthesis becomes uncoupled from DNA unwinding and lagging-strand synthesis, resulting in a ssDNA gap left behind the RF. This gap can be filled by multiple postreplicative mechanisms: (f) Strand transfer, where homologous recombination occurs within the gap, forming HJ intermediates that are resolved into cross-over (CO) or noncross-over (NCO) products; (g) Template switching, in which the nascent lagging strand serves as a template for synthesis across the gap, yielding NCO products; or (h) postreplicative TLS, where a specialized TLS DNAP fills the gap. Two main mechanisms of fork remodeling have been proposed: (c) in the RF reversal mechanism, the fork regresses as the nascent leading and lagging strands anneal, placing the lesion into a dsDNA context, and forming a HJ intermediate; and (d) nascent leading-strand DNA synthesis resumes, using the nascent lagging strand as a template *via* template switching. The reversed fork can be cleaved by HJ resolvases (i), or restored (j), followed by HR-dependent, PriA-mediated replication restart. Alternatively, TLS DNAPs bypass the lesion using the damaged template, though mutations may be introduced (e), and replisome resumes replication (k). Note that in fork remodeling pathways, the lesion is no longer present, implying that the template damage was removed by a specialized repair mechanism prior to the resumption of DNA replication. Template leading strands are shown in dark blue, template lagging strands in light blue, nascent leading strands in black, and nascent lagging strands in brown. Blue arrows indicate repair mechanisms that occur distal to the RF, while red arrows indicate those that take place at the RF. See text for detailed descriptions of the proteins involved in each pathway.

Bacteria, which lack nuclear compartmentalization and cannot spatially or temporally separate DNA replication from transcription, exhibit a remarkable ability to sense, respond to, and adapt to various endogenous and exogenous stresses that interfere with the progression of the replisome (Fig. 1(a)). These stresses arise from multiple sources, and activate specific signaling pathways triggering interconnected local and/or global responses. These responses include the repriming of DNA synthesis ahead of the lesion to continue DNA replication (Fig. 1(b)). This lesion skipping mechanism leaves stretches of single-stranded DNA (ssDNA) behind the advancing RF that need to be converted to duplex DNA in a postreplicative manner mainly *via* error-free homology-based DDT subpathways (Fig. 1(f) and (g)) (reviewed in Marians 2018, Fujii and Fuchs 2020, Cox et al. 2023, and references therein). If the DNA lesion persists, a broader global response is activated, including error-prone DDT subpathways that involve translesion synthesis (TLS) DNA polymerases (DNAPs) (Fig. 1(h)). These enzymes can bypass the lesion directly, relying on the flexibility of their active sites to accommodate damaged bases, but lead to the accumulation of mutations (Goodman and Woodgate 2013, Marians 2018, Fujii and Fuchs 2020). The lesion skipping mechanism described in *Escherichia coli* was challenged by studies analysing functions required for the recovery of stalled forks in *Bacillus subtilis* (Mangiameli et al. 2017a, Huang et al. 2023, Stoy et al. 2023). These findings suggest that several alternative pathways, which rely on replisome disassembly, operate at stalled RFs. These include error-free DDT subpathways, which remodel the stalled fork, enabling the newly synthesized sister strand to serve as a damage-free template for bypassing the lesion (Fig. 1(c) and (d)). If the DNA lesion persists, both local and broader global responses are activated, and error-prone TLS DNAPs then catalyse nucleotide incorporation—often inaccurately—opposite damaged templates at the stalled fork, allowing replication to resume (Fig. 1(e)) (reviewed in Browning and Merrikh 2024, Carrasco et al. 2024, and references therein).

The mechanisms and proteins involved in response to RS are generally conserved across species. However, each species employs distinct specific pathways to overcome replication barriers, as bacteria have evolved distinct strategies to adapt to their complex environments. Concretely, we will present the surveillance mechanisms that efficiently overcome various types of RS in two model bacteria that diverged evolutionarily >2000 million years ago. They are the best-characterized bacterium of the Proteobacteria (*a.k.a*. Pseudomonadota) phylum, *E. coli*, and of the Firmicutes (*a.k.a*. Bacillota) phylum, *B. subtilis. E. coli* is an aerobic and facultative anaerobic organism commonly found in the lower intestine and urinary tract that is thought to be physiologically uniform. In contrast, *B. subtilis* is an aerobic soil bacterium also adapted to live in the intestinal tract and the rhizosphere. *B. subtilis* differentiates into distinct subpopulations, including single motile cells and long, nonmotile chained cells. Additionally, under severe nutritional stress, *B. subtilis* can undergo multiple forms of reversible differentiation and development, such as sporulation and natural competence, forming haploid, nonreplicating cells—capabilities absent in *E. coli* (Stragier and Losick 1996, Chen and Dubnau 2004, Kearns and Losick 2005). How different DDT subpathways are coordinated, how mechanisms evolved to maintain genomic integrity in these phylogenetic distant model bacteria, and how they are intricately interconnected remains poorly understood.

In this review, we highlight similarities and species-specific differences to cope with RS. To navigate this complex topic, we first compare how cells respond to RS produced by endogenous threats before examining that induced by exogenous stressors. We explore the dynamic contributions of distinct recombinational repair mechanisms tailored to the nature of the encountered threat. Specifically, we compare the proteins involved in sensing and regulating the DNA damage responses, those that skip or remodel stalled forks, those that circumvent or bypass replication barriers through different DDT subpathways, and factors that facilitate replication restart, together with the molecular basis of their mode of action. As shown in Tables 1 (including replication genes relevant for this review) and 2 (including genes necessary to overcome RS), in which key molecular players are highlighted, while orthologous genes exist, they may encode proteins with different activity. Moreover, some alternative pathways are missing, some functions are essential, or certain proteins are absent, in one bacterium but not in the other, and in some cases, missing proteins are replaced by structurally related or unrelated alternatives. Proteins involved in reconnecting broken ends, such as those mediating non-homologous end joining (NHEJ) or resecting dsDNA ends, are not discussed. For a detailed review of DSB repair mechanisms, we refer readers to recent literature (Ayora et al. 2011, Lenhart et al. 2012, Kowalczykowski 2015, Michel et al. 2018, Amundsen and Smith 2023).

**Table 1.** Replication genes involved in overcoming RS in *E. coli* and *B. subtilis* (functional orthologs/analogs).

Activity	*E. coli*	*B. subtilis*	Role of gene product
Replication initiation	*dnaA*	*dnaA*	Response to changes in replication status
Replicative helicase	*dnaB*	*dnaC*	Hexameric DNA helicase
Helicase loader	*dnaC*	*dnaD–dnaB–dnaI*	Helicase loader and remodeler
Primosome	*dnaB*	*dnaC*	5′→3′replicative DNA helicase
	*dnaG*	*dnaG*	DNA primase
	No	*dnaE* ^b^	Family-C DNAP, extends RNA primers
Replicative DNAP HE	*dnaE* ^b^ *–dnaQ–holE* ^a^	*polC*	Family-C DNAP
	*dnaXZ* ^a,c^	No	Clamp loader subunit
	*dnaX*	*dnaX*	Clamp loader subunit
	*holA*	*holA*	Clamp loader subunit
	*holB*	*holB*	Clamp loader subunit
	*holC* ^a^	No	Clamp loader subunit
	*holD* ^a^	No	Clamp loader subunit
	*dnaN*	*dnaN*	Processivity sliding clamp
ssDNA binding	*ssb*	*ssbA*	Single-stranded binding protein
Accessory helicase	*rep* ^a^	No	3′→5′DNA helicase
Accessory DNAP	*polB* ^a^	No	Family-B, Pol II, 3′→5′ proofreading
DNA topology	*topA*	*topA*	Topo I, nicks and rotates the DNA strands
	*topB* ^a^	*topB* ^a^	Topo III, decatenase
	*gyrAB*	*gyrAB*	Topo II, cleaves and rotates both strands
	*parCE*	*parCE*	Topo IV, cleaves and rotates both strands
TLS DNAPs	*polB* ^a^	No	Family-B, Pol II, 3′→5′ proofreading
	*dinB* ^a^	*polY1* ^a^	Family-Y, PolY1/Pol IV
	*umuC* ^a^–*umuD*^a^	*polY2* ^a,d^	Family-Y, PolY2/Pol V
	No	*polA* ^a,e^	Family-A, PolA, 5′→3′exonuclease
	No	*dnaE* ^b^	Family-C, primosome component
Replication restart	No	*priA* ^f^ *–dnaD–dnaB*	Preprimosome complex, loads DnaC–DnaI
	*priA* ^f^–*priB*–*dnaT*^a^	No	Preprimosome complex, loads DnaB–DnaC
	*priA* ^f^–*priC*–*dnaT*^a^	No	Preprimosome complex, loads DnaB–DnaC
	*priC*-*rep*^a^	No	Preprimosome complex, loads DnaB–DnaC

a Nonessential function.

b DnaE/α (DnaE1), in concert with DnaQ/ε and HolE/θ, forms the error-free Pol III core enzyme, whereas *B. subtilis* DnaE (DnaE3) is an error-prone DNAP that elongates the short RNA primer synthesized by DnaG to create a hybrid RNA–DNA primer, and may act as a TLS DNAP.

c In *E. coli*, a translational frameshift on the *dnaXZ* gene produces a short DnaXZ/γ and the full-length DnaX/τ protein, whereas only full-length DnaX is present in *B. subtilis*.

d PolY2, which shares homology with the UmuC subunit of Pol V, is an active enzyme (Patlan et al. 2018).

e PolA, which shares 41% sequence identity with essential Pol I*_Eco_*, lacks a proofreading domain, and works in concert with the TLS DNAPs PolY1 and PolY2.

f PriA is a ubiquitous preprimosomal protein.

**Table 2.** Genes necessary to overcome RS in *E. coli* and *B. subtilis* (functional orthologs/analogs).

Activity	*E. coli*	*B. subtilis*	Role and activity of gene product
RecA mediators	*ssb* ^a^	*ssbA* ^a^	Negative RecA mediator, ssDNA binding
	*recO*	*recO*	Positive RecA mediator, anneals ssDNA
	*recR*	*recR*	Positive RecA mediator
	*radA* ^b^?	*radA* ^b^	Positive RecA mediator, binds branched DNA
RecA modulators	*recF*	*recF*	Positive RecA modulator, ATPase
	*rarA*?	*rarA*	Positive RecA modulator, ATPase
	*dinI*	No	Positive RecA modulator
	*recX*	*recX*	Negative RecA modulator
	No	*recU* ^c^	Negative RecA modulator
	No	*recD2*	Negative RecA modulator, 5′→3′ DNA helicase
	*uvrD*	*pcrA* ^a^	Negative RecA modulator, 3′→5′ DNA helicase
	*rdgC*	No	Negative RecA modulator
Central player	*recA*	*recA*	Recombinase, strand exchange, RS checkpoint
RecA sensors	*lexA*	*lexA*	Repressor of SOS response
	No	*disA*	RF checkpoint, c-di-AMP synthesis
Cell cycle checkpoint	*sulA*	No	Cell division checkpoint
	No	*yneA*	Cell division checkpoint
Fork remodelers	*radA* ^b^ *?*	*radA* ^b^	5′→3′ DNA helicase
	*yoaA*-*holC*	No	5′→3′ DNA helicase
	No	*recD2*	5′→3′ DNA translocase, branch migration
	*radD*	No	Processes branched intermediates
	*recQ*	*recQ, recS*	3′→5′ DNA helicase(s)
	*rep*	No	3′→5′ DNA helicase
	*priA*	*priA* ^a^	3′→5′ DNA helicase
	*ruvAB*	*ruvAB*	3′→5′ DNA translocase, branch migration
	*recG*	*recG*	3′→5′ DNA translocase, branch migration
HJ resolvase	*ruvC*	*recU* ^c^	HJ resolvase
Trafficking conflicts	(No)	*helD* ^d^	Binds and removes RNAP, (3′→5′ DNA helicase)
	*dksA*	No	Binds and removes RNAP
	*mfd*	*mfd*	Binds and removes RNAP
	*uvrD*	*pcrA* ^a^	Binds and backtracks RNAP, 3′→5′ DNA helicase
	*rapA*	*ywqA*	Binds and backtracks RNAP, ATPase
	*dinG* ^e^	*ypvA*?	5′→3′ DNA helicase
	No	*dinG* ^e^	3′→5′ exo(ribo)nuclease, ATPase
	No	*rnjA*	5′→3′ exoribonuclease
	*xni?*	*fenA*	Flap 5′→3′ exonuclease
	*rnhA*	*rnhC*	Ribonuclease at RNA:DNA hybrids

Some genes appear in multiple entries in this table because they perform distinct roles in the RS response. ?, the predicted activity has not been documented.

a Essential functions.

b RadA/Sms has two activities: unwinding DNA in the 5′→3′ direction and acting as a specialized RecA mediator at stalled forks (Torres et al. 2019a, Carrasco et al. 2024). In *E. coli*, RadA facilitates RecA-mediated DNA strand exchange, but no DNA helicase activity has been associated with it (Cooper and Lovett 2016).

c RecU, which has no homology to RuvC*_Eco_*, has two activities: cleaving HJ DNA and functioning as a RecA modulator (Carrasco et al. 2005, Cañas et al. 2008).

d HelD has limited sequence identity with HelD*_Eco_* (*a.k.a*. helicase IV), which is a weakly processive 3′ → 5′ DNA helicase, with no reported role in RNAP removal.

e DinG is a helicase with 5′→3′ DNA polarity in *E. coli*, showing limited sequence identity to the *B. subtilis* DinG 3′→5′ exo(ribo)nuclease.

## Responses to RS induced by endogenous sources

Both model bacteria employ various mechanisms to overcome endogenous threats. These include reactive oxygen species generated during cellular metabolism, which can oxidize nucleobases, and are efficiently recognized and repaired by base excision repair (BER) on duplex DNA (Baute and Depicker 2008, Wozniak and Simmons 2022). The replicative DNAP may also misincorporate ribonucleotide triphosphates (rNTPs) at a very low frequency, and these misincorporated ribonucleoside monophosphates (rNMPs) are inefficiently proofread (Evans et al. 2008). Such rNMPs are recognized and removed by ribonucleotide (RER) and nucleotide (NER) excision repair on duplex DNA (Schroeder et al. 2015, Vaisman and Woodgate 2015). These specific repair mechanisms fall outside the scope of this review, and readers are referred to comprehensive reviews for further details (Modrich and Lahue 1996, Kunkel and Erie 2005, Jiricny 2006, Baute and Depicker 2008, Vaisman and Woodgate 2015, Li et al. 2018, Wozniak and Simmons 2022, Selby et al. 2023).

If left unrepaired, these endogenous barriers can impede the progression of DNA replication. The replisome may also collide with an array of RNA polymerases (RNAPs) transcribing highly expressed genes, generating replication–transcription conflicts (RTCs), with R-loops; and so on (Lindahl 1993, Friedberg et al. 2005, Yao et al. 2013, Pham et al. 2022). Both model bacteria utilize distinct mechanisms to overcome these challenges. These barriers cause transient fork stalling and trigger local RS responses in a subset of cells, but rarely induce a global SOS response, though the mechanism modulating this response remains unclear (Friedberg et al. 2005, Kreuzer 2013).

Under unperturbed conditions, transient ssDNA regions—coated by single-stranded binding proteins (*E. coli* SSB or *B. subtilis* SsbA)—arise as a natural consequence of lagging-strand discontinuous DNA synthesis (Okazaki et al. 1968). However, neither *E. coli* nor *B. subtilis* triggers a RS response to these structures (Okazaki et al. 1968), suggesting that if recombination proteins indeed travel with the replisome *via* interaction with SSB/SsbA (Lecointe et al. 2007, Bonde et al. 2024), their activity is under a still poorly understood control.

The functions required to overcome endogenous threats include replication and transcription accessory proteins, as well as those involved in recombinational repair (Tables 1 and 2). We will first examine the local cellular responses to DNA damage caused by endogenous threats, and then focus on how cells specifically manage with the challenges posed by transcription on replication.

### Protein assembly at spontaneously stalled forks and at locations distal from replisomes in *E. coli* cells

In this bacterium, the replication machinery at each sister fork splits as it tracks along the DNA, although remaining sufficiently close to midcell. Upon replication termination, the two forks converge into a single focus before separating toward the quarter positions (Bates and Kleckner 2005, Reyes-Lamothe et al. 2008). The sites where RF velocity slows down do not coincide with the locations of highly transcribed regions, such as the *rrn* operons (Huang et al. 2023). The dynamics of replisome components have been investigated using single-molecule fluorescence imaging in unperturbed, exponentially growing cells, as well as through *in vitro* studies with reconstituted replisomes. These studies showed that Pol III* [the replicative Pol III DNAP lacking the DnaN/β-sliding clamp (the slash between DnaN and β denotes alternative names)] is replaced on a timescale of few seconds (3–6 s), the DnaN/β-sliding clamp remains associated for 30–36 s, and the replicative DnaB helicase, which overcomes leading-strand barriers under physiological conditions, is highly stable at RFs (>10 min) (Beattie et al. 2017, Lewis et al. 2017, Spinks et al. 2021). *In vitro*, when replicative Pol III holoenzyme (HE) (see Table 1) encounters a template barrier, it stalls and transiently disengages, and uncouples from DnaB, which continues unwinding the DNA, but at a significantly reduced speed (∼10-fold) (Kim et al. 1996, O’Donnell 2006). *De novo* repriming ahead of the barrier allows Pol III reengagement downstream to restart replication (Fig. 1(b)) (Heller and Marians 2006a). The lesion-containing gap left behind is circumvented or bypassed by postreplicative mechanisms (Fig. 1(f)–(h)), and must be converted to duplex DNA for specialized repair. This lesion skipping model was first proposed by Howard-Flanders and colleagues, who observed that in cells defective in NER, low-level UV irradiation did not block DNA replication (Howard-Flanders et al. 1968, Rupp and Howard-Flanders 1968). Moreover: (i) the number of replisome foci/cell remain relatively constant, but the number of Pol III HEs/cell increases post-UV treatment, though they do not colocalize with DnaB (Ghodke et al. 2019, Soubry et al. 2019); (ii) there is little coordination between leading- and lagging-strand synthesis (Graham et al. 2017, Tuan et al. 2022); and (iii) fork remodeling, which competes with lesion skipping, plays a less significant role, and is triggered upon replisome collision with the transcription machinery (Xia et al. 2016, Cox et al. 2023).

The ssDNA regions exposed by DnaB unwinding are susceptible to nucleolytic attack if not coated by SSB (Bonde et al. 2024). Spatio-temporal analysis of SSB in unperturbed wt or Δ*recB* cells suggest that DSB repair is relatively rare under unperturbed growth conditions (Cherry et al. 2023). Live-cell fluorescence microscopy studies reveal that >90% of the fluorescently labeled SSB appear as relatively dull foci, with about half colocalizing with the DnaQ/ε replisome marker (Cherry et al. 2023). However, the inherent resolution limitations of microscopy may obscure SSB binding at postreplicative gaps (in the range of 1–2 kb) that form behind the replisome (Cherry et al. 2023).

Single-molecule imaging studies reveal the existence of distinct RecA subpopulations in unstressed cells. In the majority of wt cells, RecA is sequestered in storage structures located outside the nucleoid (Renzette et al. 2005, Lesterlin et al. 2014), but in ∼20% of cells RecA forms spontaneous foci within the nucleoid, suggesting the presence of endogenous threats and RS (Renzette et al. 2005, Ghodke et al. 2019). In cells with spontaneous foci in the nucleoid, two distinct subpopulations of RecA* (*i.e*. RecA in its adenosine triphosphate (ATP) bound form, RecA·ATP, bound to ssDNA, hereafter referred to simply as RecA) are observed: (i) ∼76% of RecA foci are spatially separated from active RFs; and (ii) ∼24% of RecA foci colocalize with replisome markers, such as DnaQ/ε (Ghodke et al. 2019). These findings suggest that RecA plays a major role in postreplication gap filling, acting after lesion skipping and separated from the initial RS local response at the stalled fork (see the section “Mechanisms of gap filling behind replisomes in *E. coli*”) (Izhar et al. 2008). The RecA foci that colocalize with stalled RFs may indicate a noncanonical role for RecA at the RF, possibly in protecting it from degradation. Alternatively, in a small subset of cells, CD collisions between the Pol III HE and RNAP elongation complexes result in DSBs, thereby requiring RecA for subsequent replication restart (Dutta et al. 2011). RecF also forms spontaneous foci that colocalize with replisome markers, such as DnaX/τ in ∼22% of both *recO*^+^ or Δ*recO* unperturbed cells (Henrikus et al. 2019). In contrast, RecO appears at locations distal from replisomes and rarely colocalizes with replisome markers. The spatio-temporal localization of RecR—which neither binds DNA nor interacts with SSB—remains unknown.

What stress signal(s) promote(s) spontaneous RecA and RecF assembly at stalled RFs? Although this remains an open question, it has been shown that transient ssDNA regions accumulate with roughly equal efficiency on both leading- and lagging-strand templates. However, the transcribed nontemplate strand contains 1.7-fold more ssDNA regions than the template strand (Pham et al. 2022). It is likely that R-loops make a contribution to endogenous RS under unstressed conditions, since they account for 12%–15% of ssDNA regions (Pham et al. 2022).

Which mediator recruits RecA at stalled forks? The vast majority of RecO foci are spatially distant from the replisome, forming with similar efficiency in *recA*^+^, Δ*recA, recF*^+^, and Δ*recF* cells, and largely independent of RecR (Henrikus et al. 2019). This suggests that RecO assembles at postreplication gaps before RecR, RecA, and RecF (reviewed in Henry and Henrikus 2021). The positive mediators RecO and RecR are necessary and sufficient to facilitate RecA nucleation onto RecOR–ssDNA–SSB complexes *in vitro* (Bell et al. 2012). In short, RecO interacts with and partially displaces SSB, and in concert with RecR, helps RecA nucleation on the ssDNA (Umezu and Kolodner 1994, Bell et al. 2012, Bonde et al. 2024). RecR increases the apparent affinity of RecF for DNA (Umezu and Kolodner 1994, Webb et al. 1997). The role of RecF, which rarely colocalizes with RecO *in vivo*, as a mediator, remains elusive (reviewed in Henry and Henrikus 2021, and references therein). One plausible model for RecA loading at stalled forks involves other mediator(s) (see the section “Lesion skipping in E. coli cells”).

Since most of RecA foci do not colocalize with the replisome (Ghodke et al. 2019), the ssDNA regions generated by DnaB helicase uncoupling rarely serve as a platform for the formation of stable RecA threads (*a.k.a*. bundles or nucleoprotein filaments). These filaments are essential for LexA autocleavage and induction of the SOS response (Little 1991, Giese et al. 2008, Jones and Uphoff 2021). Consequently, spontaneous graded SOS induction occurs in only a small subset of cells: <2% of total unperturbed cells exhibit increased expression of RecA, and <0.1% of cells exhibit increased expression of TLS DNAPs or the cell division inhibitor SulA/SfiA (Courcelle et al. 2003, Friedberg et al. 2005, Kreuzer 2013). We propose that while RecA foci formation at stalled RFs and at lesions left behind the replisome is crucial for the cell to respond to RS, the timely downregulation of dynamic RecA filament growth is equally vital. RecA filament growth is regulated by positive (RecF) and negative (RecX, UvrD) modulators (reviewed in Bell and Kowalczykowski 2016, Henry and Henrikus 2021). If negative modulators predominate, they should suppress RecA filament growth to downregulate SOS induction and prevent RecA from initiating unnecessary recombination at stalled RFs. The genes encoding for negative modulators, as RecX, which passively inhibits RecA filament extension (Drees et al. 2004), or UvrD, which actively displaces RecA nucleoprotein filaments (Petrova et al. 2015), are part of the early SOS response. Consistent with this, overproduction of RecX and DinI prevents SOS induction (Yasuda et al. 1998, Stohl et al. 2003).

RecA functions not only in homologous recombination and SOS induction, but also as a component of the mutasome, since the TLS DNAP PolV (*a.k.a*. UmuCD) is activated by RecA (Jiang et al. 2009). Single-molecule experiments revealed that, under unperturbed conditions, fluorescently labeled TLS DNAPs, such as Pol IV (*a.k.a*. DinB) or Pol V are not detected (Robinson et al. 2015, Thrall et al. 2017, Henrikus et al. 2018a). This suggests that in the absence of SOS response induction, the levels of damage-inducible Pol II (*a.k.a*. PolB), Pol IV, and Pol V are insufficient to compete with Pol III for association with other replisome components (Indiani et al. 2005, 2009, Dohrmann et al. 2016, Tuan et al. 2022). Under these conditions, RecA foci likely function primarily in error-free postreplicative gap repair (Cox et al. 2023). Thus, during postreplicative gap-filling, DNA lesions are predominantly circumvented *via* substrate remodeling mechanisms, such as strand transfer (Fig. 1(f)) or template switching (Fig. 1(g)) (see the section “Mechanisms of gap filling behind replisomes in *E. coli*”). These pathways relocate the lesion onto duplex DNA, where it can subsequently be removed by excision repair mechanisms, including BER, NER, or RER (Friedberg et al. 2005).

To investigate the cellular response to a protein roadblock, a site-specific replication barrier was constructed using the TetR–YFP repressor bound to 240 tandem copies of the *tetO* operator (*tetO*_240_) (Possoz et al. 2006). Single-cell analyses revealed that induction of TetR expression efficiently and persistently stalled RF progression on the chromosome arm where the block was placed, while allowing replication of the other arm to proceed normally (Possoz et al. 2006). The force exerted by the replicative DnaB helicase on supercoiled DNA is unable to dislodge this engineered roadblock. As a result, the replisome failed to overcome the local TetR-*tetO*_240_ barrier, leading to persistent replisome stalling, and a >1000-fold decrease in cell viability (Possoz et al. 2006, Reyes-Lamothe et al. 2008, Weaver et al. 2019). Here, cell division is inhibited without SOS induction, and RecA is not observed at the site even after 2 h of roadblock induction. Controlled release of TetR binding enabled rapid replication restart independently of RecA (Possoz et al. 2006), indicating that the replisome can persist at or near a blocking nucleoprotein complex without triggering a distress signal and disassembling. This suggests that lesion skipping rarely occurs at this protein roadblock. Indeed, replacing wt DnaB with a DnaB*ts* mutant variant to enforce synchronous replisome dissociation at a nonpermissive temperature resulted in Holliday junction (HJ) accumulation and a decrease in Y-structure intermediates, which indicate that blocked RFs have undergone reversal (Weaver et al. 2019). The ability to reverse (also termed regress) stalled RFs was impaired in Δ*recQ*, Δ*recG*, or Δ*ruvAB* strains, with RecQ playing a dominant role in fork remodeling (Weaver et al. 2019). *In vitro*, RecQ converts stalled forks into reversed forks with low efficiency (Bagchi et al. 2018), RecG reverses stalled forks lacking or containing gaps in the leading-strand (McGlynn et al. 2001), and RuvAB preferentially unwinds DNA in the opposite direction to that required to form a HJ and reverses stalled forks with low efficiency (McGlynn and Lloyd 2001a, b).

### Protein assembly at spontaneously stalled forks in *B. subtilis*

In cells grown in rich medium, DNA replication proceeds largely discontinuously. Live-cell studies revealed that the replisome undergoes spontaneous disassembly ∼5 times per cell cycle, followed by reassembly once the barrier is overcome (Mangiameli et al. 2017a). In fact, when replication restart is impeded for approximately one doubling time by PriA depletion, the percentage of cells with two replicative DnaC foci—indicative of the two replisomes loaded at *oriC* and unaffected—drops to ∼13% (Mangiameli et al. 2017a). The replisome instability is primarily attributed to transcription, and consistent with this, spontaneous slow-downs in fork velocity have been observed and they coincide with the location of highly expressed *rrn* loci (Huang et al. 2023). Moreover, the replicative PolC enzyme cannot accommodate damaged templates caused by endogenous reactive oxygen species or unremoved misincorporated rNMPs (Lenhart et al. 2012).

PolC HE foci localize exclusively at mid-cell (single nucleoid) or symmetrically in each cell half (two nucleoids), with both replisomes remaining relatively close to each other for ∼80% of the replication cycle (Mangiameli et al. 2017b, Lemon and Grossman 1998). Single-molecule stoichiometry analyses suggest that while PolC and a subpopulation of DnaX are replaced every few seconds, another DnaX subpopulation exhibits a much longer dwell time than the PolC core enzyme (Liao et al. 2016, Liu et al. 2019). Live-cell fluorescence microscopy studies in exponentially growing, unperturbed cells show that the mediator protein RecO, and the preprimosomal protein PriA colocalize with replisomal markers, a colocalization absent in *ssbA*ΔC35 mutant cells (a SsbA mutant variant lacking the last 35 codons). This suggests that at least PriA and RecO travel with RFs (Lecointe et al. 2007, Costes et al. 2010). It remains unknown whether the recombination proteins RecG, RecD2, RarA, RecS (*via* YpbB), RecQ, and RecJ travel with the active replisome or are instead recruited by SsbA at stalled or collapsed RFs (Lecointe et al. 2007, Costes et al. 2010).

RecA—expressed from its native locus and promoter—is predominantly cytosolic and dispersed over the nucleoid rather than forming storage structures outside the nucleoid (Simmons et al. 2007). RecA forms spontaneous foci on the nucleoid in ∼15% of total *lexA*^+^ and *lexA*(Ind^−^) (bearing a noncleavable *lexA* mutant variant) cells (Simmons et al. 2007). These spontaneous RecA foci arise due to endogenous RS, as their formation significantly decreases upon DnaA and DnaN depletion, which compromises replication initiation (Simmons et al. 2007). Among cells with spontaneous RecA foci, >85% of them colocalize with DnaX (Simmons et al. 2007, Li et al. 2019). Furthermore, ChIP-seq analyses revealed that RecA is enriched at *rrn* loci and at sites of engineered RTCs (Million-Weaver et al. 2015a).

RecA fails to form foci in Δ*recO* or Δ*recR* cells, but does form foci in the absence of RecF or RarA (Kidane et al. 2004, Manfredi 2009, Lenhart et al. 2014, Romero et al. 2020), suggesting a specific order of protein assembly, and supporting the classification of RecO and RecR as mediators and of RecF and RarA as modulators. *In vitro* assays have shown that SsbA binds to ssDNA with very high affinity, creating a significant kinetic barrier to RecA·ATP nucleation (Carrasco et al. 2008, Manfredi et al. 2008). However, SsbA interacts with and recruits RecO onto ssDNA, which in turn promotes RecA·ATP nucleation and filament growth on SsbA–ssDNA–RecO complexes *in vitro* (Manfredi et al. 2010, Yadav et al. 2012, Carrasco et al. 2015). The RecR mediator forms large condensed nucleoprotein complexes that alter bridging distances (Alonso et al. 1993, Ayora et al. 1997a, b). RecA·ATP, with the help of mediators and positive modulators, forms nucleoprotein filaments (Carrasco et al. 2024). Conversely, the negative modulators RecX, RecU, PcrA, and RecD2 regulate RecA·ATP nucleoprotein filament length by promoting RecA disassembly through different mechanisms *in vitro*: RecU acts passively, RecX operates *via* a mixed mode, and PcrA and RecD2 actively dismantle the filaments (Le et al. 2017, Serrano et al. 2018, Carrasco et al. 2022, Ramos et al. 2022). This way, they prevent RecA from provoking unnecessary DNA recombination and SOS induction. In fact, RecA spontaneous foci rarely develop into RecA threads, which are essential for SOS induction and DNA strand exchange, and spontaneous SOS induction is observed in <1% of total unstressed cells (Simmons et al. 2009). We propose that, at sites of endogenous RS, RecA·ATP, with the assistance of mediators and positive modulators, polymerizes on ssDNA, but the negative modulators compete with positive modulators and inhibit RecA filament growth, thereby limiting SOS induction. Then, RecA at the stalled RF can recruit damage checkpoints and fork remodelers to promote fork processing through error-free DDT mechanisms (fork reversal, template switching). However, if error-free DDT subpathways become overwhelmed, the replicative PolC DNAP is replaced with TLS DNAPs (see the section “Fork remodeling and lesion bypass at stalled forks in *B. subtilis*”).

Three nonessential TLS DNAPs—PolY1 (*a.k.a*. YqjH), PolY2 (*a.k.a*. YqjW), and PolA (*a.k.a*. Pol I)—all involved in error-prone DDT subpathways, may contribute to overcome endogenous lesions at stalled forks (Sung et al. 2003, Duigou et al. 2004, 2005, Carvajal-Garcia et al. 2023). The role of the essential primosomal and error-prone DNAP DnaE in TLS remains elusive. PolY1 and PolA are constitutively expressed and physically interact with each other (Duigou et al. 2005). Furthermore, PolA interacts with DnaN and RecA (Duigou et al. 2005, Carrasco et al. 2025). Single-cell analyses using fluorescently labeled PolY1 or PolA reveal that ∼28% and ∼30% of these molecules, respectively, are static and enriched at or near RFs, through their interaction with DnaN *via* their sliding-clamp binding motif (Hinrichs and Graumann 2024, Marrin et al. 2024). It is likely that PolY1 and PolA, which lack proofreading activity, form a bipartite TLS DNAP complex that generates spontaneous mutations, bypassing the barrier through error-prone DDT at the stalled RF (Duigou et al. 2005, Carvajal-Garcia et al. 2023). It remains unknown whether PolY1 and PolA travel with the replisome during processive replication or instead they associate with proteins assembled at stalled forks (*e.g*. RecA). Finally, RecA assembled at stalled forks contributes to loading preprimosomal proteins to promote replication restart (Million-Weaver et al. 2015a).

To investigate the cellular response to a protein roadblock, an engineered site-specific replication barrier was constructed using the TetR repressor bound to 120 tandem copies of the *tetO* operator (*tetO*_120_). The roadblock (TetR-*tetO*_120_ complex) efficiently and persistently stalls replication, while leaving replication of the other arm intact (Bernard et al. 2010). Upon TetR repressor expression, ∼60% of cells exhibited RecA foci 45 min after induction of the replication roadblock, inhibition of cell division occurred through alternative pathways, and the SOS response was not induced (Bernard et al. 2010). The response to such RS is mediated, in part, by DnaA, which alters the expression of ∼56 genes (42 upregulated) (Goranov et al. 2005, Bernard et al. 2010). Controlled release of TetR repressor binding resulted in a significant delay (∼40 min) before replication restart (Bernard et al. 2010), suggesting that the replicative DnaC helicase cannot overcome the local barrier, and the replisome may be dislodged, as described at RTCs (Mangiameli et al. 2017a).

Following spontaneous RS, many recombination proteins (RecA, RecO, RecR, RecF, RarA, PcrA, and RnhC) primarily assemble at stalled RFs (Romero et al. 2019a, Simmons et al. 2007, Manfredi 2009, Merrikh et al. 2015, Hinrichs and Graumann 2024). Whether they also localize to sites distal from the replisome remains unclear. Therefore, lesion skipping and postreplication repair of lesion-containing gaps left behind the advancing replisomes are likely minor pathways, if they occur at all. Furthermore, RecA colocalizes with DnaD and DnaC at engineered RTCs, and is required for replication restart in response to this local RS (Million-Weaver et al. 2015a, Merrikh et al. 2011).

## The impact of transcription on RS

In bacteria, transcription is a major source of endogenous RS, since replication and transcription are not temporally or spatially separated (reviewed in Mirkin and Mirkin 2005, Merrikh et al. 2012, Browning and Merrikh 2024). As DNA-tracking machineries, both the replisome and RNAP translocate along the DNA, generating (+) supercoils ahead and (−) supercoils behind them (Liu and Wang 1987, Wu et al. 1988, Hiasa and Marians 1996, Wang 2002). The fast-moving replicative DNAP can successfully bypass a slow-moving RNAP elongation complex transcribing low- to moderately expressed genes, regardless of whether they are in codirectional (CD) or head-on (HO) orientation. During replisome passage, RNAP is retained within the transcription bubble, allowing RNA synthesis to resume rapidly without an apparent fitness cost under unstressed conditions (French 1992, Liu et al. 1993, Huang et al. 2023). However, highly expressed regions on the same template strand (CD orientation), such as ribosomal operons (*rrn* loci), exhibit high RNAP occupancy (arrays of RNAPs) that may lead to RS (Huang et al. 2023). When DNAP collides with an array of RNAPs in CD orientation in unperturbed *B. subtilis* cells, or when replisome-driven supercoiling encounters transcription-driven supercoiling at regions of high RNAP density in HO orientation in *E. coli* and *B. subtilis* cells, topological and/or steric constraints halt the progression of both moving machines (reviewed in Mirkin and Mirkin 2005, Merrikh et al. 2012, Lang and Merrikh 2018, and references therein). In both model bacteria, essential and highly expressed genes are predominantly encoded on the leading-strand to prevent HO collisions (Rocha and Danchin 2003, Merrikh et al. 2011).

The underwound state generated by transcription-induced (−) supercoiling behind RNAP may promote the reannealing of displaced nascent mRNA with the complementary DNA strand, leading to the formation of stable RNA–DNA hybrids with a displaced ssDNA strand, named R-loops (reviewed in Mirkin and Mirkin 2005, Merrikh et al. 2012, Drolet and Brochu 2019, Browning and Merrikh 2024, and references therein).

The molecular machineries responsible for transcription elongation and mRNA translation are highly conserved between both model bacteria, although some differences exist. The *E. coli* RNAP core enzyme consists of four distinct subunits (α_2_ββ′ω) (Masse and Drolet 1999), whereas *B. subtilis* RNAP (α_2_ββ′ωδε) core enzyme contains two additional small subunits (δ and ε) that influence RNAP recycling (Lane and Darst 2010).

### RNAP transcription elongation in *E. coli*

As in most bacteria, *E. coli* maintains a transcription elongation rate that tightly matches ribosome’s speed under various growth conditions, including shifts in carbon sources and growth-phase transition; coupling transcription and translation (Proshkin et al. 2010, McGary and Nudler 2013, Kohler et al. 2017). The lead ribosome and the elongating RNAP core enzyme are bridged by the essential transcription factors NusG and NusA, which bind at the interface of both machines, stabilize the RNAP-ribosome tandem and contribute to maintain productive transcription–translation coupling (O’Reilly et al. 2020, Wang et al. 2020a, Webster et al. 2020). This coupling, along with ribosome trailing, prevent RNAP backtracking, reduce the likelihood of R-loop formation by limiting exposure of the RNA 5′-end to invade the (−) supercoiled DNA behind RNAP, facilitate forward RNAP movement at nucleoid periphery, and avoid premature mRNA release when cells are grown in rich Luria-Bertani (LB) medium (Lane and Darst 2010, Proshkin et al. 2010, Nudler 2012, Kohler et al. 2017, Johnson et al. 2020, Wang and Artsimovitch 2021). In fast-replicating cells, ∼68% of all transcribing RNAPs are dedicated to rRNA synthesis (Condon et al. 1995). When untranslated *rrn* loci are transcribed, there are no ribosomes behind to accelerate the slow-moving RNAP. Here, RNAP core enzyme is modified by essential antitermination complexes (including NusA, NusB, NusE/RpsJ, and NusG), to prevent Rho-dependent termination of rRNA transcription. This antitermination complex likely takes the role of the leading ribosome in accelerating transcription (Roberts 2010, Washburn et al. 2020, Webster et al. 2020, Wang and Artsimovitch 2021).

In the presence of template lesions or protein barriers, RNAP stalls and pauses transcription, respectively. Meanwhile, ribosomes moving along the emerging mRNA prevent RNAP backtracking and R-loop formation (Proshkin et al. 2010, Nudler 2012). Accessory factors as DksA, Rho, RapA, and GreAB interact with RNAP to form ternary complexes that alter the conformational states of RNAP, helping preserve genome integrity. Rho and RapA contribute to the control of cytotoxic R-loops *in vivo*, as the growth defects observed in their absence can be rescued by controlled expression of RnhA (Leela et al. 2013, Brewer et al. 2025). RapA backtracks, while DksA removes, RNAP from nucleic acids (Liu et al. 2015, Myka et al. 2019). Upon backtracking, the RNAP catalytic site disengages from the RNA 3′-end rendering the RNAP elongation complex inactive. GreA and GreB then facilitate RNAP-mediated cleavage of the nascent RNA to restore the RNA 3′-OH in the active site (Abdelkareem et al. 2019).

### RNAP transcription elongation in *B. subtilis*

There is a clear gene organization bias that minimizes gene transcription from the lagging-strand template (Rocha and Danchin 2003, Wang et al. 2007a, Merrikh et al. 2011). Fork-velocity measurements in exponentially growing unperturbed cells reveal that transcription of low- to moderately expressed genes in CD or HO orientation does not significantly affect replisome speed. However, an array of RNAPs transcribing the highly expressed *rrn* operons in the CD orientation cause transient replisome slow-downs (*i.e*. pauses) (Huang et al. 2023) and, in ∼40% of cells, the replisome spontaneously disassembles when cells are grown on rich medium (Mangiameli et al. 2017a). A severe conflict occurs when an engineered RTC in HO orientation inhibits RF progression and the stalled RF is reversed (Stoy et al. 2023)

Unexpectedly, the elongating RNAP core enzyme moves twice as fast as ribosomes, leading to transcription–translation uncoupling (Johnson et al. 2020, Zhu et al. 2021). Indeed, transcription and translation occur within distinct cellular domains: transcription is localized to the nucleoid, while ribosomes are positioned near the cell poles (Lewis et al. 2000, Mascarenhas et al. 2001). Due to transcription–translation uncoupling, ribosome-free nascent RNAs may be prone to forming hairpins and R-loops, promoting RNAP backtracking. Additionally, there is no mechanism for removing RNAP from transcribing mRNAs containing premature stop codons.

Many of the functions that, in *E. coli*, induce transcription antitermination, reduce RNAP backtracking, and help to cope with RTCs are either absent (as DksA and GreB), dispensable (as NusG, NusB, and Rho), restricted to rRNA synthesis [NusB and NusE (RpsJ or S10)], play an opposite activity (NusG promotes pause-free rRNA synthesis in *E. coli* but induces RNAP pausing in *B. subtilis*) or travel with the elongating RNAP (as *B. subtilis* NusA and GreA) (Krasny and Gourse 2004, Johnson et al. 2020, Webster et al. 2020, Yakhnin et al. 2020, Wang and Artsimovitch 2021, Driller et al. 2023). NusG, which interacts with the nontranscribed strand to induce RNAP pausing at T-rich tracts, stimulates NusA-dependent intrinsic termination (Johnson et al. 2020, Webster et al. 2020, Yakhnin et al. 2020, Wang and Artsimovitch 2021). Rho stimulates ∼10% of intrinsic terminators (Mandell et al. 2022), and a Δ*rho* mutation has minimal impact on mRNA synthesis (Wang and Artsimovitch 2021). GreA associates with RNAP at the secondary channel and assists the nucleolytic activity of the RNAP core or HE to restore the RNA 3′-OH in the active site, indirectly rescuing a backtracked RNAP (Kusuya et al. 2011, Roghanian et al. 2011). Finally, the essential transcription factor NusA, which plays a major role in termination (at ∼77% of intrinsic terminators) (Mandell et al. 2022), has a minor role in RNAP pausing compared to NusG (Jayasinghe et al. 2022). Notably, NusA depletion increases the expression of key replication and repair genes, including *polC, dnaB, dnaD, dnaI, priA, recG, disA*, and *radA* (Mondal et al. 2016). It remains largely unclear whether RNAP stalling at RTCs leads to ribosome collisions and how this may ultimately compromise cellular fitness.

### Specific responses to RTCs and R-loops in *E. coli*

One can wonder whether R-loops are pathological structures or not. For instance, they can play beneficial roles in RnhA (*a.k.a*. RNase HI)-dependent DNA replication initiation of specific episomal elements without triggering RS (Itoh and Tomizawa 1980), and in LexA-dependent or LexA-independent host replication initiation in Δ*rnhA* mutant cells (reviewed in Kogoma 1997, Drolet and Brochu 2019). However, R-loops may also interfere with DNA replication elongation.

In unstressed wt cells, replisome and transcription elongation-related unwinding create topological challenges, as the movement of the replisome or RNAP through the DNA accumulates (+) supercoils ahead and (−) supercoils behind (Liu and Wang 1987, Wu et al. 1988, Hiasa and Marians 1996, Wang 2002) that can lead to R-loop accumulation. To maintain proper DNA supercoiling, cells encode nucleoid associated proteins (HU, IHF, H-NS, and so on), which impose DNA topological constrains, as well as topoisomerases, which resolve topological problems associated with DNA transactions such as supercoils, catenates, tangles, and knots (Bates and Kleckner 2005, Macvanin and Adhya 2012, Dame et al. 2020). Among them, Topo I (*a.k.a*. TopA) interacts with RNAP and is localized genome-wide in association with actively transcribing RNAPs (Sutormin et al. 2022), Topo II (*a.k.a*. GyrAB) enriches near RFs and downstream of highly transcribed operons (Stracy et al. 2019), Topo III (*a.k.a*. TopB) forms foci that colocalize with the RF (Koster et al. 2010, Lee et al. 2019, Kim and Guo 2024), and Topo IV (*a.k.a*. ParCE) is distributed uniformly throughout the nucleoid (Zawadzki et al. 2015). It seems that in unstressed wt cells, R-loop homeostasis is maintained by regulating its formation and degradation: by relaxing transcription-induced (−) supercoiling behind the moving RNAP, topoisomerases prevent R-loop formation (Brochu et al. 2018, Drolet and Brochu 2019, Stolz et al. 2019).

**Figure 2.**
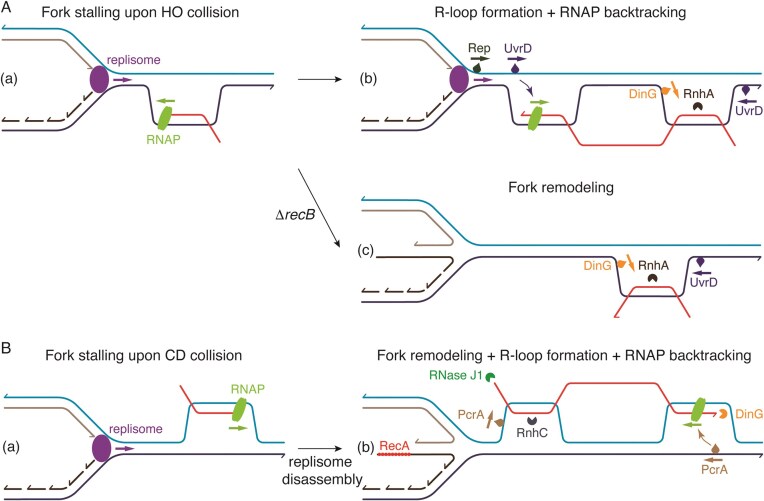
Mechanisms of stalled RF rescue in response to HO RTCs in *E. coli* (A), or CD RTCs in *B. subtilis* (B). Accessory proteins that aid in resolving RTCs are illustrated. Other proteins may also contribute to overcome RTCs, but for the sake of simplicity, they are not depicted here (see text). Colored arrows indicate the directionality of each enzyme’s activity. (AB-a) The transcribing RNAP (light green) impedes the progression of the replisome (purple). (A-b) Replisome-RNAP collisions facilitate the formation of an RNA–DNA hybrid with a displaced ssDNA segment—an R-loop—behind. The Rep (dark green) and UvrD (dark blue) helicases, translocating in the 3′→5′ direction, may backtrack the RNAP. The helicase DinG (orange) or UvrD may displace the RNA strand by translocating in opposite directions. The endoribonuclease RnhA cleaves the RNA moiety of the RNA–DNA hybrid. (A-c) In the absence of RecB, the fork is remodeled. (B-b) The topologically constrained region between the moving replisome and RNAP promotes RTC and R-loop formation. The stalled replisome disassembles, a fork remodeler enzyme reverses the stalled fork, and RecA may coat the nascent lagging-strand to protect it. RecA, as a hub, loads DDT functions to utilize the 3′-end of the nascent strand as a template for DNA synthesis. The RNase J1 (green) exonuclease degrades RNA with 5′ monophosphates in 5′→3′ direction. The helicase PcrA (light brown) assembles on the leading-strand template and displaces the RNA. The stalled RNAP interacts with and loads PcrA, which translocates in the 3′→5′ direction along the template lagging-strand, and could backtrack the RNAP. The endoribonuclease RnhC (blue) cleaves the RNA moiety of the RNA–DNA hybrid. The exo(ribo)nuclease DinG (orange) processes the displaced RNA in the 3′→5′ direction.

*In vitro* reconstitution assays revealed that when a replisome encounters short R-loops on the lagging-strand template (HO orientation), they have little impact on RF progression. The DnaB helicase can translocate over RNA–DNA duplexes, and the Pol III HE can displace the RNA strand as during Okazaki fragment synthesis (Pomerantz and O’Donnell 2008, Brüning and Marians 2021). Fork stalling increases with the number of RNAPs transcribing a given region or with the length of R-loops (Fig. 2A) (Brüning and Marians 2020). *In vivo*, SSB bound to ssDNA interacts with and may recruit the RnhA ribonuclease, which degrades the RNA moiety of R-loops, and the RecG and DinG helicases, which displace the RNA–DNA hybrids (reviewed in Cox et al. 2023, Bonde et al. 2024). Indeed, mutant strains lacking *rnhA, dinG, recG, rep*, or *uvrD* accumulate R-loops (Kogoma 1997, Boubakri et al. 2010, Fonville et al. 2010, Bonde et al. 2023, Cox et al. 2023). Δ*rnhAB* mutant cells accumulate UV-induced RTCs at the *rrn* operons, which inhibit RF progression (Kouzminova et al. 2025). This RTCs accumulation is suppressed by RNAP mutations that reduce the stability of transcription complexes (rpoB* or rpoC*) (Kouzminova et al. 2025). As a Δ*rnhA* mutant is synthetically lethal in the Δ*recG* context (Hong et al. 1995, Bonde et al. 2023), and RnhA overexpression suppresses the Δ*dinG* synthetic lethality at engineered HO RTCs when cells are grown in rich medium (Boubakri et al. 2010), it could be reasoned that DinG, and/or RecG can substitute for RnhA in removing R-loops associated either with RTCs or with stalled RNAPs. Furthermore, Rho-dependent termination is essential solely for reducing R-loop occurrence across the genome, with R-loop lethality linked to RTCs, as the lethality of Δ*rho* or Δ*nusG* mutants in rich-medium is rescued by expression of the R-loop-helicase UvsW, which removes the reannealed nascent untranslated transcripts, or suppressed by the RpoB* mutation (McGlynn et al. 2012, Nudler 2012, Leela et al. 2013).

The transiently stalled replisome may load accessory helicases or nucleases to remove the barrier *via* protein–protein interactions. DnaB interacts with and may recruit Rep (Guy et al. 2009), and a poorly defined replisome subunit recruits UvrD, even under unperturbed conditions (Wollman et al. 2024). *In vitro*, Rep and UvrD, both ancillary SF1 DNA helicases with 3′ → 5′polarity, displace RNAP clashed in CD or HO orientation, with UvrD promoting RNAP backtracking and displacing RNA–DNA hybrids (Epshtein et al. 2014, Hawkins et al. 2019, Syeda et al. 2019). Additionally, in the absence of RNAP, a naked R-loop serves as a substrate for UvrD, which can unwind the DNA strand of RNA–DNA hybrids, whereas Rep cannot (Brüning and Marians 2021). The rpoB* or rpoC* RNAP mutations suppress the synthetic lethality observed in Δ*rep* Δ*uvrD* or Δ*rep* Δ*uvrD* Δ*dinG* mutants (Boubakri et al. 2010). SSB can also serve as a recruiting factor, as it interacts with accessory helicases such as DinG and RecG, the RnhA ribonuclease, and, together with HolC/χ, interacts with and recruits the YoaA helicase (reviewed in Bonde et al. 2024, and references therein). The RnhA nuclease removes the RNA strand of R-loops (reviewed in Browning and Merrikh 2024). The contribution of the YoaA–HolC/χ complex, another SF2 helicase, which moves in the 5′→3′ direction, is unknown (Weeks-Pollenz et al. 2023).

RNAP may also contribute, by protein–protein interaction, to the recruitment of UvrD, Mfd, DksA, RapA, GreAB, NusG, and Topo I, proteins involved in processing stalled RNAPs or R-loops (Toulme et al. 2000, Cheng et al. 2003, Trautinger et al. 2005, Epshtein 2015, Liu et al. 2015, Abdelkareem et al. 2019, Myka et al. 2019, Brüning and Marians 2021). Mfd, a transcription-repair coupling factor, acts as a forward translocase, binds to stalled RNAPs, dislodging them from sites of DNA damage and releasing the truncated transcripts independently of replication (Park et al. 2002, Pomerantz and O’Donnell 2010, Le et al. 2018, Ho et al. 2020). GreA and GreB rescue backtracked RNAPs by facilitating the cleavage of a short (GreA, <3-nt) or a longer (GreB, >3-nt) nascent RNA, restoring the RNA 3′-OH group in the active site to reactivate backtracked RNAPs (Toulme et al. 2000, Abdelkareem et al. 2019). RapA, a backward translocase, rescues RTCs by promoting RNAP backtracking and subsequent removal (Liu et al. 2015, Inlow et al. 2023, Brewer et al. 2025). The essential transcription factors NusG, NusA, and DksA also prevent RTCs (O’Reilly et al. 2020, Wang et al. 2020b, Webster et al. 2020). An interplay between replication- and the transcription-recruited enzymes has been observed, since the Δ*holC* mutation is colethal with Δ*dksA* or Δ*nusA*, and this lethality is suppressed by RpoB* or RpoC* mutations (Trautinger et al. 2005, Cooper et al. 2021).

Engineered strains with a reduced number of *rrn* operons exhibit a high RNAP occupancy at the remaining *rrn* operons, leading to strong CD RTCs. In response to such RS, cells extend the “lag phase”, allowing replication to transiently increase the relative copy number of *rrn* operons and partially alleviate growth defects (Fleurier et al. 2022). A reduced number of *rrn* operons/genome limits the effect of transcription–translation coupling and ribosome trailing but indirectly enhances RNAP backtracking, leading to an increase in DSBs at RTCs (Proshkin et al. 2010). Indeed, cells containing only a single *rrn* operon per chromosome exhibit RNAP relocation to the remaining *rrn* operon, persistent replisome stalling, and increased R-loop formation due to excessive (−) supercoiling at the overtranscribed *rrn* operon. This leads to SOS induction and a ∼2800-fold increase in cell death when grown in LB medium, while 56% of cells still form colonies in minimal medium (Fleurier et al. 2022, Fan et al. 2023). The lag phase is further extended and cell death increases (>2000-fold) in the single *rrn* operon strain when mutations such as Δ*recA*, Δ*recB*, Δ*rnhA*, Δ*rapA*, Δ*greA*, Δ*mfd*, Δ*ruvA*, or *lexA*3(Ind^−^) are introduced. These cells also exhibit increased SOS response, leading to elevated mutagenesis (Fleurier et al. 2022). The absence of RecF, which is involved in lesion skipping, had no effect in cells with a single *rrn* operon (Fleurier et al. 2022). Given that the RecBCD complex is the only recombination complex essential for cell viability under conditions of heightened HO RTCs (De Septenville et al. 2012), we hypothesize that in the overtranscribed *rrn* operon, RecBCD processes either the regressed arm of a reversed RF (resembling a one-ended DSB) or two-ended DSBs resulting from CD RTCs or excessive R-loop formation (Dutta et al. 2011, Fleurier et al. 2022). At this CD RTC RnhA removes the R-loop, RapA, GreA, and Mfd process the backtracked RNAP, and RuvAB could remodel branched DNA intermediates (Selby and Sancar 1994, Dutta et al. 2011, De Septenville et al. 2012, Liu et al. 2015, Abdelkareem et al. 2019). Notably, introducing the rpoC* mutant allele (which results in a reduction of transcription initiation), the *lexA*(Def) allele (which derepresses SOS induction), or overexpressing RnhA or UvsW, significantly decreases lag phase and mitigates RS in these cells with a single *rrn* operon (Fleurier et al. 2022).

The InvA and InvBE strains, in which the *rrnA* or *rrnBE* operon(s), respectively, is(are) inverted (Boubakri et al. 2010), have been used to identify proteins involved in overcoming HO RTCs. Replisome collisions with an array of RNAPs transcribing the highly expressed *rrnA* or *rrnBE* operons(s) in HO orientation lead to fork remodeling and reduce cell viability in Δ*recB* (by >5000-fold) and Δ*dinG* (by >1000-fold) cells when plated on LB agar (Boubakri et al. 2010, De Septenville et al. 2012). Analysis of InvA or InvBE DNA in Δ*recB* cells revealed evidence of fork reversal, and the accumulation of reversed forks is not prevented in Δ*recA*, Δ*recG*, or Δ*ruvAB* cells (De Septenville et al. 2012), provided that DSBs were not formed. *In vitro* studies, however, have shown that: (i) the RecG or RecQ translocase interacts with branched DNA intermediates, pushing the stalled fork backward and reannealing the nascent template strands, forming reversed forks (also known as regressed forks), which are protective HJ-like structures; (ii) the RecG or RuvAB translocase migrates the HJ-like structure in the opposite direction, leading to fork restoration; (iii) the PriA DNA helicase displaces the lagging-strand, facilitating the reloading of the replicative DnaB helicase; and (iv) the RuvAB–RuvC or RecG–RuvC complex resolves, or the RecQ–Topo III complex dissolves, the reversed forks, leading to a one-ended DSB (Fig. 2A) (reviewed in McGlynn and Lloyd 2002, Kowalczykowski 2015, Michel et al. 2018, Bianco and Lu 2021, Amundsen and Smith 2023, Cox et al. 2023, and references therein). The precise enzyme(s) responsible for fork remodeling in the InvA and InvBE strains remains unclear (Fig. 2A) (De Septenville et al. 2012). Decreasing *rrn* operon expression (with the *rpoC* Δ215–220 mutation), significantly improves the viability of cells with inverted *rrn* operons grown on LB agar (De Septenville et al. 2012). Similarly, destabilization of ternary RNAP complexes *via* the rpoB* or rpoC* mutation enhances cell viability upon RTCs (Trautinger et al. 2005, Dutta et al. 2011). Since RnhA overexpression fails to restore viability of Δ*recBC* InvBE cells, it is assumed that R-loop accumulation does not contribute to cell death in this genetic background. Additionally, the absence of Rep or UvrD aggravates the requirement for RecBCD in maintaining viability on LB agar (De Septenville et al. 2012). Indeed, the Δ*dinG*, Δ*dinG* Δ*rep*, or Δ*dinG* Δ*uvrD* mutations strongly compromises the plating efficiency on LB agar in the inverted *rrn* operons strain. Other studies showed that RnhA overexpression rescues the viability of Δ*dinG invBE* and Δ*topA* cells (Masse and Drolet 1999, Boubakri et al. 2010). These findings suggest that: (i) in cells with inverted *rrn* operons grown on LB agar DinG or RnhA removes R-loops (Boubakri et al. 2010); (ii) RecBCD processes remodeled forks (De Septenville et al. 2012); (iii) Rep (or UvrD) backtracks RNAP and facilitates DNA damage repair; and (iv) the role of RecA remains largely unclear.

### Specific responses to RTCs and R-loops in *B. subtilis*

Unlike in *E. coli*, the replisome undergoes transient pausing at highly transcribed regions (*e.g. rrn* loci), as revealed by quantitative locus-specific measurements of fork velocity in exponentially growing, unstressed wt cells (Huang et al. 2023). Many of the functions required to resolve RTCs in *E. coli* are either absent (*e.g*. Rep, GreB, DskA, and YoaA–HolC/χ), dispensable (*e.g*. NusG, NusB, and Rho), or have different activities (*e.g*. DinG functions as an exonuclease rather than a DNA helicase, and NusG induces RNAP pausing), in *B. subtilis*. Furthermore, *B. subtilis* possesses functions absent in *E. coli* [*e.g*. RecD2, HelD, RnjA (*a.k.a*. RNaseJ1)] (Carrasco et al. 2024). Additionally, the PolC HE cannot utilize the RNA strand of R-loops for repriming DNA synthesis (Sanders et al. 2010, Seco and Ayora 2017). PolC extends the hybrid RNA–DNA primers synthesized by the DnaG–DnaE primase–polymerase complex (Sanders et al. 2010, Seco et al. 2013). A specialized “primase” that synthesizes a DNA primer, as PrimPol, is absent (Bianchi et al. 2013, García-Gómez et al. 2013). These differences suggest that distinct proteins may contribute to resolving RTCs and R-loops in these two model bacteria.

Replisome- and transcription elongation-related topological challenges can be resolved by nucleoid associated proteins—including essential Hbsu and dispensable EbfC, Rok, and LrpC—as well as by topoisomerases—including the essential (or synthetically lethal) GyrAB, ParCE, and TopA; and the dispensable TopB (Berkmen and Grossman 2006, Macvanin and Adhya 2012, Dame et al. 2020, Hirsch and Klostermeier 2021, Karaboja and Wang 2022). ParE and ParC localize uniformly throughout nucleoids in ∼86% of cells, GyrAB forms foci that frequently colocalize with DnaX, and TopA displays two distinct localization patterns: in 66% of cells, it is diffusely distributed across the nucleoids, whereas in 34% of cells, it forms distinct foci, which frequently colocalize with the SMC complex but not with DnaX (Tadesse and Graumann 2006). The localization of TopB has not been studied. Topological constraints contribute to the detrimental effects of HO RTCs: conditional depletion of either GyrAB or ParCE is deleterious to cells experiencing engineered HO RTCs, and transiently increases replisome stalling (Lang and Merrikh 2021), suggesting that GyrAB or ParCE-mediated (−) supercoiling promotes R-loop formation. Indeed, in cells lacking RnhC, which resolves R-loops, inhibition of GyrAB or ParCE reduces R-loop levels and alleviates R-loop-induced replisome stalling at HO genes (Lang and Merrikh 2021).

Protein–protein interaction studies have been used to investigate how cells could recruit proteins to RTCs. In cells growing under unperturbed conditions, RNAP and RecA physically interact and may function as recruiting hubs (Carrasco et al. 2024). RecA physically interacts with PcrA, RnhC, and DinG (Carrasco et al. 2025). RNAP interacts with several translocases (as Mfd, YwqA, PcrA, and HelD) that contribute to RNAP recycling, or with enzymes that may remove, or degrade the RNA strand of R-loops (such as PcrA, RnhC, GreA, and RnjA) (Delumeau et al. 2011, Mondal et al. 2016, Sanders et al. 2017, Carrasco et al. 2024). RNAP-binding proteins have been mechanistically linked to overcoming RTCs and removing R-loops, but not to R-loop formation (Carrasco et al. 2024). The roles of RecA, Mfd, YwqA, PcrA, HelD, RnhC, GreA, and RnjA in this process have been recently revisited (Fig. 2B) (reviewed in Carrasco et al. 2024, and references therein).

PcrA is mainly bound at sites of RTCs as the *rrn* operons (Merrikh et al. 2015). Lethality induced by PcrA depletion is suppressed by *recA* inactivation or by mutations in *rpoB* or *rpoC*, which reduce RNAP–DNA complex stability (Yeesin 2019, Moreno-Del Alamo et al. 2020). Moreover, PcrA depletion severely compromises cell viability in the Δ*rnhC* or Δ*dinG* background, and the Δ*recA* mutation is synthetically lethal in the Δ*rnhC* background. We hypothesize that RecA, in concert with RecO, prevents R-loop accumulation rather than promoting RNA–DNA hybrid formation, as was proposed for *E. coli* RecA (Kasahara et al. 2000, Zaitsev and Kowalczykowski 2000). RnhC, which interacts and may travel with RNAP even in the absence of exogenous DNA damage, is the main enzyme in removing the RNA portions of R-loops (Lang et al. 2017, Schroeder et al. 2023, Carrasco et al. 2025). In fact, RNA–DNA hybrids accumulate at *rrn* operons in Δ*rnhC* cells (Schroeder et al. 2023), as well as at engineered HO RTCs (Stoy et al. 2023). The DinG enzyme is a 3′→5′ exo(ribo)nuclease capable of removing R-loops *in vitro* (McRobbie et al. 2012, Carrasco et al. 2025). Live cell studies have shown that both RnhC and DinG spontaneously associate with replication markers, and a frequent association with RFs occurs upon exogenous threats (Hinrichs and Graumann 2024, Carrasco et al. 2025). In addition to RnhC and DinG, other proteins may contribute to R-loop removal: *in vitro*, RNase J1, which is an endo- and 5′→3′ exoribonuclease, degrades the nascent RNA and disassembles the stalled RNAP *via* a “torpedo” mechanism (Fig. 2B) (Sikova et al. 2020). PcrA removes the RNA strand of RNA–DNA hybrids *in vitro* (Moreno-Del Alamo et al. 2021).

Transcription–translation uncoupling should facilitate RNAP backtracking and R-loop formation, and the *rrn* operons are hotspots for CD RTCs in rapidly growing cell populations (Merrikh et al. 2011, Johnson et al. 2020, Huang et al. 2023). In a fraction of rapidly growing cells, RecA forms spontaneous foci that colocalize with DnaX and associate with HO RTCs, as revealed by ChIP analyses (Million-Weaver et al. 2015b, Simmons et al. 2007). In turn, DnaX foci colocalize with rRNA loci in unperturbed cells (Merrikh et al. 2011). In a RecA-dependent manner, preprimosomal DnaB and DnaD, and the DnaC replicative helicase accumulate at forks stalled by a HO conflict to promote replication restart (Million-Weaver et al. 2015a). This suggests that once RecA assembles at the RTC, it protects the stalled fork, and upon interacting with RNAP works as a hub to overcome spontaneous RS, orchestrate DDT and facilitate replication restart (reviewed in Browning and Merrikh 2024, Carrasco et al. 2025). Indeed, preprimosomal DnaD colocalizes with CD conflicts at *rrn* operons and at engineered HO collisions, and this association is reduced in Δ*recA* cells (Million-Weaver et al. 2015a). Since DnaC association with engineered conflict regions increased significantly following PcrA depletion in both *recF*^+^ and Δ*recF* cells (Million-Weaver et al. 2015a, Merrikh et al. 2015), and RecA filaments are disassembled from ssDNA by PcrA *in vitro* (Carrasco et al. 2022), we assume that noncanonical RecA activities may contribute to overcoming RTCs, facilitating replication restart.

The functions required to overcome CD RTCs were also analysed constructing an engineered strain with decreased number of *rrn* operons. A progressive reduction in the number of *rrn* operons leads to a graded increase in RNAP occupancy on the remaining operons. When a critical threshold is exceeded, CD RTCs occur, with the most severe effect observed when 9 of the 10 *rrn* operons are deleted (Condon et al. 1995, Fleurier et al. 2022). In the absence of exogenous threats, cells with only one *rrn* operon (*i.e. rrnA* operon at the *oriC* region) exhibit high RNAP occupancy, an extended lag phase to increase the number of available *rrn* operons by replication, increased R-loop accumulation, and a ∼10-fold decrease in viability, compared to the wt strain. Remarkably, a *B. subtilis* strain with a single *rrn* operon is significantly more resilient that an *E. coli* strain with a single *rrn* operon (Fleurier et al. 2022).

To evaluate the impact of HO RTCs on cell physiology, engineered strains with highly expressed genes on the lagging strand were constructed: (i) by ectopically relocating the *oriC* region to the 257° chromosomal position, thereby forcing an array of RNAPs to transcribe the *rrnB* operon in HO orientation; and (ii) by inverting the *rrnI, rrnH*, and *rrnG* operons at the *oriC* region, generating the *rrnIHG*-inversion strain (Srivatsan et al. 2010, Yeesin 2019). Single-cell analyses revealed that highly expressed genes in the HO orientation destabilize the replisome, with >80% of cells containing only 6 rather than 12 DnaC protomers in the replication factory (Mangiameli et al. 2017a). RecA forms foci in ∼97% of these cells, suggesting replisome disassembly and RecA-mediated replication restart (Srivatsan et al. 2010). The reversed *rrnIHG* operons are tolerated in *lexA*^+^ or *lexA*(Ind^−^) strains grown in minimal medium, leading to SOS induction in <2% of cells, and cell death in up to 1% of total cells (Srivatsan et al. 2010, Yeesin 2019). Conversely, they are deleterious in wt cells grown in LB; these cells fail to replicate their chromosomes, resulting in a >1700-fold decrease in plating efficiency (Srivatsan et al. 2010, Yeesin 2019). In *rrnIHG*-inversion cells, deletion of *recF* or the presence of the *lexA*(Ind^−^) mutation had a milder effect on viability (2- to 4-fold compared to the *rrnIHG*-inversion in wt cells), while inactivation of *recO, recR, recA, ruvAB, recU*, or *rnhC* significantly reduced the viability (∼30-fold), and deletion of *addB* resulted in a ∼90-fold decrease in viability upon plating on LB agar (Yeesin 2019). Overexpression of RnhC did not improve viability, while overexpression of PcrA increased plating efficiency by ∼20-fold and reduced the presence of RecA foci to ∼75% of cells, in *rrnIHG*-inversion cells grown in LB (Yeesin 2019). Mutations in RNAP (*rpoB* N475T or *rpoC* A755P), which destabilize transcription complexes, suppressed the deleterious effect of the *rrnIHG*-inversion in viability on LB agar plates. Thus, at least RecO, RecR, RecA, AddB, RuvAB, RecU, and RnhC are crucial for overcoming HO RTCs, whereas the role of RecF and SOS response remains unclear (Yeesin 2019). At HO conflicts, RecA in concert with preprimosomal proteins promotes replisome reassembly (Million-Weaver et al. 2015a).

## Proteins required to survive RS in response to exogenous threats

A variety of exogenous agents can induce DNA lesions. These include bulky lesions caused by UV [or its mimetic 4-nitroquinoline-1-oxide (4NQO)], which are sensed, recognized, and repaired on duplex DNA by global genomic NER or transcription-coupled repair; and the nonbulky alkylating lesions induced by methyl methanesulfonate (MMS), which are efficiently detected and removed from duplex DNA by BER. These specialized repair mechanisms fall outside the scope of this review. Readers interested in these pathways are directed to comprehensive reviews (Baute and Depicker 2008, Wozniak and Simmons 2022, Selby et al. 2023).

If unrepaired, these lesions on the DNA template trigger both local and global responses, as the replicative DNAP cannot accommodate unremoved bulky or nonbulky lesions. When replisomes stall at a distorted or damaged base, they may either skip the lesion or undergo fork remodeling (Marians 2018, Cox et al. 2023, Carrasco et al. 2024). As a by-product, template lesions on ssDNA cannot be repaired by specialized pathways (Friedberg et al. 2005, Kreuzer 2013), and they need to be placed on a duplex DNA to be removed.

Some proteins that contribute to overcoming exogenous threats in *E. coli* are either absent in *B. subtilis*, such as Rep, Pol II, DinI, RdgC, ExoI, DksA, YoaA–HolC, PriB, DnaT, PriC, or RadD, or have a different activity, such as DinG, HelD, TopB, or RadA/Sms. On the contrary, some proteins (RecU, RecD2, DisA, or RnjA) are present in *B. subtilis*, but not in *E. coli*. Additionally, some proteins (Hbsu, PriA, DnaB, DnaD, or PcrA) are essential in *B. subtilis*, but not in *E. coli* (Tables 1 and 2). This suggests that at least some mechanisms for resolving RS differ between these two model organisms.

### Proteins required in *E. coli*

The proteins required to overcome a RS induced by exogenous threats have recently been revisited (reviewed in Cox et al. 2023, and references therein). These proteins are categorized as follows: (i) replisome and repair hubs, including DnaB, SSB, and RNAP (Kim et al. 1996, Johnson and O’Donnell 2005); (ii) proteins targeting stalled forks, such as RecF, RecQ, RecG, RarA, RuvAB, RuvC, Rep, UvrD, DinG, YoaA–HolC/χ, RadD, RecA, and so on (Courcelle and Hanawalt 2003, Michel et al. 2018, Cox et al. 2023); (iii) proteins modulating DNA topology (as Topo I, Topo II or Topo IV, and RNAP), and compaction (including the nucleoid associated proteins: HU, IFH, H-NS, and so on) (Wang 2002, Courcelle and Hanawalt 2003, Kowalczykowski 2015, Dame et al. 2020, Cox et al. 2023); (iv) proteins acting at lesion-containing gaps left behind replisomes, such as ExoI, RecJ, RecQ, RecO, RecR, RecA, RadD, RecX, DinI, RuvABC, and so on (Michel et al. 2018, Cox et al. 2023); (v) proteins involved in fork remodeling after transient replisome disassembly, including RecG, RecQ, RuvAB, RadA, and RadD (Michel et al. 2018, Cox et al. 2023); (vi) proteins acting on persistent ssDNA at lesion-containing gaps upon SOS response induction, such as the TLS DNAPs Pol II, Pol IV, and Pol V (Goodman and Woodgate 2013, Marians 2018, Fujii and Fuchs 2020, Cox et al. 2023), and (vii) proteins facilitating replication restart after transient replisome disassembly, including PriA, PriB, DnaT, PriC, and Rep (Heller and Marians 2006a, Michel and Sandler 2017, Windgassen et al. 2018).

### Proteins required in *B. subtilis*

A straightforward approach to identifying proteins crucial for overcoming RS under exogenous threats involves damaging the DNA of mature, inert haploid spores, defective in one or more DNA repair protein(s), and then synchronously reviving these predamaged spores under unstressed conditions. Ionizing radiation induces dose-dependent DNA lesions, including damaged template bases, single-strand nicks, and two-ended DSBs (reviewed in Setlow and Christie 2023, and references therein). In dormant haploid spores, these lesions are processed after spore germination (t0 min to t15 min post-spore revival) during the ripening stage (t15 min to t60 min) by: (i) BER to remove damaged bases; (ii) LigD-dependent or LigD-independent pathways to repair single-strand nicks; and (iii) Ku (*a.k.a*. YkoV)- and LigD-dependent non-homologous end joining (NHEJ) to reconnect two-ended DSBs (Weller et al. 2002, Wang et al. 2006, de Ory et al. 2016, Setlow and Christie 2023, Pospisil et al. 2024). During the early outgrowth stage (t60 to t80 min)—which precedes the onset of DNA replication at *oriC*2—proteins required for DNA replication and recombination are synthesized (Sinai et al. 2015, Swarge et al. 2020, Pospisil et al. 2024).

When ionizing radiation induces high levels of template base damage or when BER is incomplete, unrepaired lesions stall DNAP elongation during early outgrowth, leading to RS. The proteins required to overcome RS are: (i) RecA; (ii) RecO and RecR mediators, and the positive (RecF, RarA) and negative (RecX, RecU, RecD2, PcrA) modulators; (iii) LexA; (iv) the DNA integrity scanning protein A, DisA, checkpoint sensor and repair licensing factor; (v) fork remodelers, including RuvAB, RecG, and RecD2 branch migration translocases, as well as the RadA/Sms DNA helicase; (vi) TLS polymerases (PolY1 and PolY2) that, in concert with PolA, facilitate template lesion bypass; and (vii) Mfd (Ayora et al. 1996, Duigou et al. 2005, Moeller et al. 2008, Vlasic et al. 2014, Raguse et al. 2017, Valenzuela-Garcia et al. 2018). The roles of other proteins that participate in overcoming RS in vegetative cells—including RNase J1 (*a.k.a*. RnjA), RnhC, HelD, and FenA (*a.k.a*. ExoR or YpcP)—in the repair of preirradiated spores during early outgrowth stage remain to be elucidated.

The AddAB nuclease–helicase complex and the RecJ ssDNA exonuclease, in concert with a RecQ-like helicase (RecQ or RecS), are crucial for long-range resection of broken DNA ends (Fernández et al. 1998, Sanchez et al. 2006). Interestingly, in the absence of both long-range end resection pathways, the predamaged spores remain recombination-proficient, and as capable of repairing preexisting DNA damages as the wt control (Vlasic et al. 2014). This suggests that recombination-mediated DSB repair is less critical for overcoming RS under haploid conditions, as it is limited by the need for an intact homologous template (Carrasco et al. 2024). Moreover, these helicases and nucleases are mainly synthesized during the later stages of spore outgrowth and upon transition to vegetative growth, when the NHEJ system is inactive or poorly operative (Keijser et al. 2007, Nicolas et al. 2012, Sinai et al. 2015, Swarge et al. 2020, Pospisil et al. 2024). However, the possible relevance of RecQ or RecS in overcoming RS during spore revival may be masked by a redundancy in their role as fork remodelers.

## Responses to DNA RS

When the replisome encounters exogenous threats, most responses involve tightly coordinated RS processes. Both model bacteria possess evasion mechanisms that are part of a stress-inducible genetic network, many of which are unrelated to DNA replication. Since these mechanisms are not primarily induced by RS, their analysis falls outside the scope of this work. We direct readers to recent general stress response reviews on transcriptional reprogramming mechanisms (Hecker et al. 2007, Price 2011, Bonilla 2020, Rodríguez Ayala et al. 2020, Bouillet et al. 2024, and references therein), protein quality control (Elsholz et al. 2017, Mahmoud and Chien 2018, Driller et al. 2023, and references therein), and persistence to host-encoded toxin or antibiotic stress (Lewis 2010, and references therein, Urbaniec et al. 2022, Salzer and Wolz 2023).

Under certain stress conditions, both bacteria utilize the second messenger (p)ppGpp to increase transcription and respond to environmental changes. In *E. coli*, (p)ppGpp directly binds to RNAP and, in coordination with the transcription factor DksA, modulates the expression of hundreds of genes (Sanchez-Vazquez et al. 2019). In *B. subtilis*, however, (p)ppGpp does not interact with RNAP, but reduces the intracellular GTP pool, thereby deactivating the CodY repressor and leading to the derepression of >200 genes (Kriel et al. 2012, Brinsmade 2017, Anderson et al. 2021). The functions controlled by (p)ppGpp are not directly linked to RS (McGlynn and Lloyd 2000, Kamarthapu et al. 2016, Anderson et al. 2021, Driller et al. 2023), and therefore their role is outside the scope of this review. For a more detailed discussion on this topic, we refer readers to recent reviews (Krasny and Gourse 2004, Kriel et al. 2012, Anderson et al. 2021, Driller et al. 2023).

### Activation of DNA damage responses in *E. coli*

UV light has been widely used to investigate the proteins involved in DNA repair. In response to a UV dose 20 J/m^2^ (800–1200 adducts/chromosome), the nucleoid becomes compacted, and DNA replication transiently pauses before restarting at later time points (Rupp and Howard-Flanders 1968, Courcelle and Hanawalt 2001). When the replicative Pol III HE encounters unremoved bulky lesions, it halts and transiently uncouples from DnaB *in vitro*. DnaB continues unwinding dsDNA, albeit at a reduced speed (Lewis et al. 2017, Spinks et al. 2021), generating the initiating molecular signal—a ssDNA region—that is rapidly coated by the SSB protein.

RecA·ATP mainly nucleates onto postreplicative gaps, as expected by the lesion skipping model (Ghodke et al. 2019). This process is aided by RecA mediators and inhibited by negative modulators (Cox et al. 2023). RecA·ATP, in a RecOR-dependent manner, nucleates onto ssDNA–SSB complexes. Subsequently, RecF promotes the dynamics of the nucleoprotein filament (RecA*), while RecX interacts with and limits RecA filament extension (reviewed in Cox 2007, Bell and Kowalczykowski 2016, Henry and Henrikus 2021, and references therein). Persisting RecA* allosterically induces the autolytic cleavage of cytosolic LexA transcriptional repressor. Upon autocleavage, LexA can no longer bind dsDNA to repress transcription, leading to the induction of a graded SOS response with the expression of genes involved in diverse pathways (early SOS genes) (Little 1991, Courcelle et al. 2001, Giese et al. 2008, Jones and Uphoff 2021, Cory et al. 2024). Indeed, inactivation of *recO, recR*, or *recF* reduces and delays SOS induction (Whitby and Lloyd 1995). In the first wave of the SOS response, genes involved in direct repair and error-free homologous recombination are induced. Among them are RecA itself, the positive (DinI) and negative (RecX, UvrD) modulators, and the branch migration translocase RuvAB (Courcelle et al. 2003, Kreuzer 2013). DinI interacts with and stabilizes RecA filaments competing with RecX activity (Lusetti et al. 2004). UvrD discourages RecA filament formation and actively displaces RecA from ssDNA (Veaute et al. 2005, Petrova et al. 2015).

In response to persistent ssDNA, oligomeric RecA* further promotes the cleavage of cytosolic dimeric LexA, shifting the equilibrium from DNA-unbound autoproteolyzed LexA toward the active expression of SOS genes with slow off-rate dissociation. Among the late SOS genes derepressed are SulA/SfiA, and TLS DNAPs (Pol II, Pol IV, and Pol V) (Courcelle et al. 2001, Friedberg et al. 2005, Kreuzer 2013). SulA/SfiA acts as a checkpoint master regulator, delaying cell division by directly inhibiting FtsZ polymerization. Meanwhile, cells activate mutagenesis through TLS DNAPs, which lack proofreading activity, introducing mutations that may enhance genetic diversity, adaptive mutation, and the evolution of antimicrobial resistance (Friedberg et al. 2005, Kreuzer 2013). Once the SOS response is turned off, SulA/SfiA is degraded, allowing cell division to resume (Friedberg et al. 2005, Kreuzer 2013). A poorly characterized RecA-dependent but LexA-independent global response has been also documented (Khil and Camerini-Otero 2002), but remains to be elucidated.

### Activation of DNA damage responses in *B. subtilis*

Live-cell microscopy has been used to analyse the players that participate in the activation of the response to different types of DNA damage or inhibition of the PolC HE. Following exposure to a very low dose of UV irradiation (1 J/m^2^, 40–60 adducts/chromosome), the exogenous threat results in the accumulation of RecA foci at stalled RFs in >85% of both *lexA*^+^ and *lexA*(Ind^−^) cells within 5 min post-UV treatment, and these type of RecA foci are not sufficient for SOS induction (Simmons et al. 2007). *In vivo*, RecO and RecR promote RecA nucleation, and the RecF and RarA positive modulators contribute to RecA filament growth (Alonso et al. 2013, Lenhart et al. 2014, Romero et al. 2020). We reasoned that at such UV doses, nucleated RecA may protect stalled forks from degradation—akin to the role of its eukaryotic homolog RAD51 (Hashimoto et al. 2010, Zellweger et al. 2015). The activity of RecO, RecR, RecF, and RarA may be insufficient to support RecA·ATP filament growth (RecA threads) at or near stalled forks, and the dynamic extension of RecA filaments may be inhibited by several negative modulators—RecX, RecU, PcrA, and RecD2—which disassemble RecA from ssDNA both *in vivo* and *in vitro*, thereby preventing SOS induction (Cañas et al. 2008, Cárdenas et al. 2012, Romero et al. 2020, Carrasco et al. 2022, Ramos et al. 2022). Except for PcrA, the expression of these modulators is independent of the SOS response (Au et al. 2005).

Upon exposure to moderate UV doses (25–40 J/m^2^) or treatment with mitomycin C, the SOS response is induced, as evidenced by the increased expression of the TagC-CFP reporter and RecA accumulation (Gassel and Alonso 1989, Au et al. 2005, Goranov et al. 2006). Under these conditions, RecA forms foci in concert with RecO and RecR in >94% of cells, with ∼85% of these foci colocalizing with stalled replisomes (DnaX) (Simmons et al. 2007, Lenhart et al. 2014). RecF and RarA also form foci that colocalize with DnaX (Kidane et al. 2004, Manfredi 2009, Romero et al. 2020). We propose that, in this scenario, persistent ssDNA regions at stalled forks or an unknown factor may be required for RecA-mediated SOS response activation. RecA·ATP nucleates on ssDNA, and the activity of positive modulators prevails over the negative modulators, resulting in RecA foci conversion into RecA threads (RecA filament growth), and subsequent SOS response induction (Gassel and Alonso 1989, Au et al. 2005, Cárdenas et al. 2012, Lenhart et al. 2014, Romero et al. 2020). RecA nucleoprotein filaments promote LexA self-cleavage, with the expression of 30–35 genes upregulated (Au et al. 2005). Notably, among the gene products involved in overcoming RS, only 6 (*recA, lexA, ruvAB, pcrA*, and *polY2*) are SOS-upregulated and shared across both model bacteria (Au et al. 2005).

Once repair is completed, RecA threads dissipate. At ∼180 min post-DNA damage, <5% of wt cells display visible RecA threads, whereas in the Δ*recX* or Δ*recD2* mutants, these structures persist for longer (Cárdenas et al. 2012, Ramos et al. 2022). RecA interacts with and may recruit RecX, RecU, PcrA, and RecD2, which inhibit RecA filament extension and facilitate disassembly, thereby contributing to SOS response shut-off (Cárdenas et al. 2012, Le et al. 2017, Serrano et al. 2018, Carrasco et al. 2022, Ramos et al. 2022). This aligns with observations that SOS induction fails in the absence of RecA mediators and is both reduced and delayed when positive modulators are missing (Gassel and Alonso 1989, Cárdenas et al. 2012, Romero et al. 2020). For instance, the absence of RarA does not impair RecA foci formation, but RecA threads are disassembled and become shorter in the Δ*rarA* background (Romero et al. 2020), suggesting that RarA acts as a positive modulator of dynamic RecA filaments.

Selective inhibition of the PolC enzyme using 6-(p-Hydroxyphenylazo)-uracil (HPUra) blocks elongation, possibly causing PolC to backtrack due to its exonuclease proofreading activity (Brown 1970). This leads to PolC dissociation from the replisome, since its exchange rate is increased ∼3-fold compared to unstressed cells, and some DnaX molecules, but not all, are also more rapidly exchanged (Liao et al. 2016, Li et al. 2019). These results suggest that some replisome uncoupling exists and that the activity of the DnaC helicase may produce ssDNA gaps. Following HPUra treatment, RecA foci are observed in >95% of cells, colocalizing with DnaX in 93%–97% of cases. Concurrently, the SOS response is induced, as evidenced by using a YneA-CFP reporter (Wang et al. 2007b, Bernard et al. 2010, Lenhart et al. 2014). HPUra treatment activates different overlapping global responses, including: (i) a genuine LexA-dependent SOS response (changes in expression of 30–35 genes) (Goranov et al. 2006); (ii) a RecA-dependent but LexA-independent global response (changes in expression of ∼100 additional genes in a background free of extrachromosomal elements) (Goranov et al. 2006); (iii) a RecA-independent but DnaA-dependent global response (altering expression of >50 genes) (Goranov et al. 2005, Ishikawa et al. 2007); and (iv) a RecA-independent but indirectly DnaA-dependent global response (∼340 genes affected), where DnaA indirectly affects *sda* gene expression, and the Sda developmental checkpoint indirectly couples cell differentiation (sporulation, biofilm formation) with RS responses (Burkholder et al. 2001, Veening et al. 2009, Washington et al. 2017).

DisA, which is absent in *E. coli*, contributes as a checkpoint to maintaining genome integrity, by monitoring DNA integrity and triggering responses to recover from RS (Bejerano-Sagie et al. 2006). In the absence of DisA, cell survival significantly decreased in response to damage-induced fork stalling (Gándara and Alonso 2015). DisA forms a highly mobile focus that scans along the chromosome searching for branched intermediates, while converting a pair of ATPs into the essential cyclic 3′, 5′-diadenosine monophosphate (c-di-AMP) (Bejerano-Sagie et al. 2006, Witte et al. 2008, Gándara et al. 2017). *In vitro*, DisA preferentially binds branched structures (Gándara et al. 2021). Upon fork stalling by MMS addition, DisA forms a discrete static focus on the nucleoid and suppresses c-di-AMP synthesis to levels comparable to the Δ*disA* context. Focus pausing is also observed in ∆*addAB* ∆*recJ* cells—blocked in end resection—suggesting that the initiating molecular signal for DisA pausing is neither the accumulation of ssDNA nor duplex DNA ends (Torres et al. 2019b, Bejerano-Sagie et al. 2006, Witte et al. 2008, Oppenheimer-Shaanan et al. 2011, Torres et al. 2019c). In the absence of RecA (or RecO), DisA fails to pause (Torres et al. 2019b). We hypothesize that RecA assembled at stalled forks interacts with and recruits DisA onto branched intermediates, suppressing c-di-AMP synthesis. Finally, low c-di-AMP level results in the accumulation of (p)ppGpp that directly inhibits DnaG activity (Wang et al. 2007a, Denapoli et al. 2013), suggesting that DisA indirectly inhibits cell proliferation to maintain genome integrity during RS.

## Mechanisms of fork reactivation

*E. coli* and *B. subtilis* employ distinct, hierarchical strategies to respond to RS. In both model bacteria, the replicative DNAP stalls in response to endogenous or exogenous threats, although the frequency and mode of response differ, suggesting that they have evolved adaptations to their specific ecological niches. In *E. coli*, endogenous threats cause transient fork stalling at a very low frequency (0.2 events/replication cycle) (Michel and Sandler 2017), whereas exogenous threats increase the frequency of fork stalling to multiple events per replication cycle. In this bacterium, it is believed that damage is simply skipped, leaving a lesion-containing gap behind the advancing replisome (Fig. 1(b)). In a second step, the gap is filled and the lesion is circumvented primarily through error-free DDT subpathways (Fig. 1(f) and (g)). If these subpathways are dysregulated, error-prone DDT subpathways bypass the lesion (Fig. 1(h)) (Marians 2018, Cox et al. 2023). Lesion skipping and postreplicative gap repair compete with fork reversal, which appears to play a lesser role in *E. coli* and is primarily triggered in response to RTCs or protein–DNA roadblocks (De Septenville et al. 2012, Weaver et al. 2019). Conversely, in *B. subtilis*, spontaneous replisome disassembly occurs at least five times per cell cycle, with this frequency increasing significantly upon exogenous threats (Mangiameli et al. 2017a). Cells appear to rely more heavily on error-free fork remodeling subpathways (one-step repair model) (Fig. 1(c) and (d)) (Stoy et al. 2023), and if these pathways are dysregulated, error-prone DDT subpathways bypass the lesion (Fig. 1(e)). Ultimately, once the damaged or distorted template base is in duplex DNA, specialized repair pathway(s) (e.g. BER, NER, RER, and so on) can remove the lesion (Friedberg et al. 2005, Kreuzer 2013).

### Lesion skipping in *E. coli* cells

Following a very low UV dose (1–2 J/m^2^), Δ*uvrA* cells synthesize the same amount of DNA as unirradiated controls, albeit with a 15–20 min delay (Rupp and Howard-Flanders 1968). This is consistent with *in vitro* data showing that when the Pol III HE encounters an exogenous leading-strand lesion, it transiently disassembles, skips the lesion, and reengages at a *de novo* reprimed region ahead to facilitate replication restart while leaving ∼500-nt long lesion-containing gaps behind the replisome, which are coated by SSB and repaired in a postreplicative manner (Fig. 1(b)) (Rupp and Howard-Flanders 1968, Yeeles and Marians 2011, Yeeles and Marians 2013). In unstressed cells, the fraction of ssDNA is similar in both the leading- or lagging-strands. Upon UV treatment, ssDNA regions increase >3-fold in number and in average length, while maintaining their relative distribution (Pham et al. 2022). These results suggest that lesion skipping occurs in response to both endogenous or exogenous threats, and in both strands. Lagging-strand reinitiation benefits from its intrinsic mechanism of DNA synthesis, and Pol III HE cycles forward and reengages with the next DnaN/β-sliding clamp, utilizing an available short RNA primer synthesized by DnaG at a new Okazaki fragment, in concert with DnaB (Pagès and Fuchs 2003, Marians 2018). In contrast, leading-strand reinitiation may require preprimosome-dependent loading of the replisome (reviewed in Heller and Marians 2006a, Yeeles et al. 2013, Marians 2018, and references therein).

The overall intensity of the SSB signal remains unchanged, though the number of foci per cell increases slightly, suggesting a relatively stable intracellular SSB concentration before and after UV treatment (Cherry et al. 2023). Additionally, upon UV irradiation, the total number of bright SSB foci increases in wt but not in Δ*recB* cells, indicating that most of these foci do not correspond to DSB repair sites. Half of the SSB foci do not colocalize with replisome markers, with the most common ssDNA species attracting SSB binding being lesion-containing gaps left behind the replisome (Cherry et al. 2023).

RecA storage structures dissolve to allow cytosolic RecA redistribution to form foci in ∼56% of total cells 60 min post-UV treatment (20 J/m^2^) (Sassanfar and Roberts 1990, Kim et al. 1996, Ghodke et al. 2019, Jones and Uphoff 2021). At this point, cytosolic RecA exists in two discrete subpopulations: one-third of RecA foci colocalize with replisome markers (as DnaQ/ε) in *lexA*^+^ or *lexA*3(Ind^−^) cells, and two-third of RecA foci assemble mainly at locations distal from the replisome in *lexA*^+^ cells (Ghodke et al. 2019). This suggests that, in most cells, lesion skipping and postreplicative gap repair occurs. RecO (and possibly RecR) promote the formation of RecA foci at sites distal from replisomes in response to both endogenous and exogenous threats, with the latter occurring in an SOS-dependent manner (Ghodke et al. 2019, Henrikus et al. 2019). At postreplicative gaps, RecA contributes to protect the DNA and to fill the gap (Cox et al. 2023). Interestingly, the role of RecA at stalled forks appears to extend beyond its canonical function in postreplicative gap repair (Cox et al. 2023). Notably, RecA does not participate in fork remodeling or in the resolution of R-loops (Boubakri et al. 2010, De Septenville et al. 2012).

RecO foci rarely colocalize with DnaX/τ within 50 min post-UV treatment, and RecF is not sufficient for RecA loading onto SSB-coated ssDNA (Henrikus et al. 2019), thus the mediator(s) that facilitate(s) RecA assembly at stalled forks remains unidentified. One hypothesis is that an unidentified mediator may displace SSB. A potential candidate for this mediator role is DprA (*a.k.a*. Smf). *B. subtilis* DprA interacts with and loads RecA onto SsbA–ssDNA or SsbB–ssDNA complexes (Yadav et al. 2013, 2014). Investigating whether RecA recruitment to stalled forks in the Δ*recO* Δ*recBCD* background depends on DprA will be of significant interest.

Live-cell imaging studies show that RecF, RecQ, and RarA form foci that colocalize with replisome markers (Sherratt et al. 2004, Henrikus et al. 2019). Specifically, RecF foci colocalize with DnaX/τ in ∼40% of cells (Henrikus et al. 2019). What roles could RecF, RecQ, RarA, and RecA play at stalled RFs? Beyond its canonical role in modulating RecA·ATP filament growth, RecF interacts with DnaN/β and DnaG, suggesting it plays a noncanonical role: RecF helps disengage the Pol III core enzyme arrested on both leading and lagging strands, and replisome reengagement upon repriming for DNA synthesis resumption (Henry et al. 2023). RecQ, in concert with RecA and SSB, may disrupt fork remodeling (Harmon and Kowalczykowski 1998). The precise function of RarA at stalled forks remains unclear. Evidence suggests it may participate in postreplication gap repair (Jain et al. 2021a, b), either by acting at RecA-generated recombination intermediates (Shibata et al. 2005) or independently of RecA (Jain et al. 2021b). Alternatively, RarA may also assist in resolving reversed forks. Indeed, a Δ*rarA* Δ*ruvB* mutant shows severe growth defects, and the Δ*recG* or Δ*recQ* mutation is synthetically lethal in the Δ*rarA* Δ*ruvB* background, a phenotype suppressed by inactivation of either *recO* or *recF* (Jain et al. 2021a). The precise function of RecA at stalled forks, provided that DSBs are not generated, remains unclear.

When the leading-strand DNAP is halted by a lesion or barrier but the replisome remains bound to DNA, preprimosomal proteins are not required for replication to resume. Leading-strand synthesis may be reinitiated downstream of the lesion in a reaction that depends on DnaG and DnaXZ/γ, but is independent of preprimosomal proteins (Yeeles and Marians 2011, 2013). Alternatively, when the DNAP stalls at CD RTCs, it can use an RNA transcript or the RNA strand of an R-loop as a primer to continue leading-strand synthesis, leaving behind a gap (Pomerantz and O’Donnell 2008, Brüning and Marians 2020). However, when DnaB disassembles, upon transient disassembly of the Pol III HE, replication reinitiation requires the loading of a new DnaC–DnaB complex by various dispensable preprimosomal proteins, followed by DnaG-mediated *de novo* repriming (reviewed in Michel and Sandler 2017, Windgassen et al. 2018, and references therein). A new Pol III HE from the cytosol can then reassemble to resume synthesis ahead of the lesion (Beattie et al. 2017, Lewis et al. 2017, Soubry et al. 2019, Henry et al. 2023). With the exception of PriA (a SF2, 3′→5′ DNA helicase), the specific preprimosomal proteins (PriB, PriC, DnaT, and Rep) are not widely conserved outside the γ-Proteobacteria Class (Windgassen et al. 2018, Bianco and Lu 2021, Blaine et al. 2023, and references therein). *In vivo* studies revealed that PriA, which is crucial for DnaB helicase loading outside *oriC*, forms spontaneous foci that colocalize with the replisome in ∼7% of exponentially growing cells. However, under exogenous stress, >70% of cells exhibit PriA foci that colocalize with replisome markers (Soubry et al. 2019). PriA exhibits a ∼2-fold higher binding affinity for 5′-fork DNA (fork with no nascent leading strand) compared to 3′-fork DNA (fork with no nascent lagging strand) (Tanaka and Masai 2006, Wang et al. 2020a).

*In vitro* reconstitution assays using purified proteins and substrates that mimic different types of stalled forks have revealed distinct mechanisms for DnaB loading outside *oriC*. First, PriC, which efficiently recognizes 5′-fork intermediates, loads Rep. Rep facilitates displacement of the nascent lagging-strand, and both in concert reload a single DnaB–DnaC complex onto the lagging-strand template (Heller and Marians 2006a, b). Second, PriA recognizes spontaneously stalled RFs with either duplex structures (*e.g*. at certain RTCs or protein roadblocks) or 3′-fork intermediates, and displaces SSB from the parental lagging-strand, exposing a binding site for PriB on the lagging-strand template (Heller and Marians 2005, Duckworth et al. 2023). Next, DnaT binds to the PriAB-3′-fork DNA complex. PriA–PriB–DnaT recruits the DnaC–DnaB complex onto the lagging-strand template *via* a ring breaking mechanism (Heller and Marians 2005, Duckworth et al. 2023). A third pathway, involving PriA–PriC–DnaT, remains less well understood, as it has not yet been reconstituted *in vitro* (Michel and Sandler 2017, Windgassen et al. 2018, Wong et al. 2021, Cox et al. 2023). Notably, recruitment of Pol III HEs at locations distal to the RF, in a DnaB-independent manner, was observed following UV treatment (Soubry et al. 2019). This may reflect gaps that require filling, with Pol III HE recruitment potentially mediated by the SSB protein.

### Mechanisms of gap filling behind replisomes in *E. coli*

Upon lesion skipping, the lesion-containing gaps left behind the replisome must eventually be filled and sealed. In fact, the number of replisome foci/cell remained relatively constant for 60 min post-UV treatment in a DnaB-dependent manner, while the number of Pol III HEs/cell and SSB/cell increases at locations distal from replisomes in a DnaB-independent manner, consistent with the two-step repair model (Ghodke et al. 2019, Soubry et al. 2019, Cherry et al. 2023).

At least four distinct postreplicative repair mechanisms for gap filling—identified primarily exposing cells to UV treatment—have been identified: (i) RecA-mediated strand transfer; (ii) RecA-mediated template switching; (iii) RecA-independent template strand transfer process(es); and (iv) gap-filling by error-prone TLS DNAPs (Courcelle and Hanawalt 2003, Izhar et al. 2008, Yeeles and Marians 2013, Marians 2018, Michel et al. 2018, Laureti et al. 2022). These mechanisms place the lesion in duplex DNA to be repaired by specialized repair systems (NER, BER, and RER). The first three pathways (Fig. 1(f) and (g)), collectively referred to as homology-directed gap repair, account for the vast majority of events (∼98%) observed *in vivo* during the repair of gapped plasmids containing various lesions within the gap (Izhar et al. 2008, Naiman et al. 2016). The latter mechanism, responsible for ∼1%–2% of DDT across template lesions, is activated when lesion bypass is delayed, resulting in persistent ssDNA regions that sustain the SOS response (Fig. 1(h)) (Ohmori et al. 2001, Friedberg et al. 2005, Izhar et al. 2008, Naiman et al. 2016). Impairing RecA loading—*via* mutations in presynaptic proteins—leads to a reduction in homology-directed gap repair and a concomitant increase in TLS (Laureti et al. 2022).

Using a plasmid-based system that allowed to discriminate between mechanisms it was observed that ∼80% of homology-directed gap repair occurs by strand-transfer and ∼20% by template-switching (Izhar et al. 2008). These mechanisms use the undamaged nascent strand as a template for DNA synthesis to circumvent the lesion in an error-free manner, generating different types of branched intermediates behind the RF. *In vitro* studies reveal that SSB bound to the ssDNA gap interacts with, and stimulates the activities of the RecJ 5′→3′ ssDNA exonuclease and the RecQ 3′→5′ DNA helicase, to enlarge the ssDNA gap at the 5′-end. SSB recruits RecO onto the SSB–ssDNA complexes (Bonde et al. 2024). Subsequently, RecO, along with RecR, partially displaces SSB facilitating RecA·ATP nucleation (Umezu and Kolodner 1994, Hobbs et al. 2007, Bell et al. 2015, Shinn et al. 2023). RecA·ATP then forms a dynamic nucleoprotein filament in concert with mediators and modulators, and initiates a homology-driven search (Bell and Kowalczykowski 2016). Upon finding homology, RecA may catalyse strand transfer or template switching by annealing the complementary strands (Izhar et al. 2008). SSB bound to the lesion-containing gap may also interact with and load HolC/χ, and indirectly recruit the Pol III core enzyme in a DnaB-independent manner (Chang et al. 2019, Soubry et al. 2019). Pol III then synthesizes DNA using the complementary nascent strand as template. A branch migration translocase, either RecG or RuvAB—or perhaps RadA—may subsequently reconstitute the RF, or, in concert with the RuvC resolvase, resolve the intermediate by cleaving the HJ-like structure into cross-over (CO) or noncross-over (NCO) products in a RecA-independent manner (Fig. 1(f)) (Gupta et al. 2014, Lovett 2017, Bianco and Lu 2021). In fact, Δ*ruvAB* exhibits synthetic lethality in the Δ*rarA* Δ*recG* or Δ*rarA* Δ*recQ* backgrounds, and Δ*recG* causes significant growth defects in the Δ*radA* background, and these phenotypes are suppressed by deletions of *recO* or *recF*, suggesting that these functions are involved in postreplication gap repair (Jain et al. 2021a, Cooper et al. 2015, Romero et al. 2020, Bonde et al. 2023).

RecA-independent template switching accounts for up to 16% of gap filling events, *via* mechanisms yet to be elucidated. Similarly, this mechanism occurs at gaps left behind the RF between repeated sequences (Lovett 2017). This mechanism involves reengaging the sister nascent strands after dissociation from the template DNA to provide a primer for DNA synthesis, possibly mediated by RarA or other DNA helicases (Jain et al. 2021b). In Δ*radD* cells, RecA-independent template switching increases by ∼100-fold (Romero et al. 2020), by a poorly unknown mechanism.

In the absence of RecA- and RarA-dependent mechanisms, it has been observed a significant increase in template switching through poorly defined alternatives (Cox et al. 2023). SSB may recruit factors to process the DNA end, and facilitate priming of DNA synthesis for gap filling. Indeed, SSB interacts with the clamp-loader subunit HolC/χ, and this subunit interacts with the YoaA helicase (Weeks-Pollenz et al. 2023). The second alternative, DnaK-dependent template-switching mechanism, which leads to rearrangements between repeated sequences, is increased in DNA replication mutants such as DnaE/α), DnaQ/ε), HolC/χ) SSB, PriA, and so on (Lovett 2017). The DnaK chaperone potentially remodels the replisome complex, facilitating the unwinding of the 3′-nascent strand to permit the annealing of the complementary strand without the need for a strand-invasion protein, such as RecA or a strand annealing protein as RecO (Lovett 2017).

The last postreplicative gap-filling mechanism involves error-prone TLS DNAPs, which lack proofreading activity (Fig. 1H) (Goodman and Woodgate 2013, Marians 2018, Fujii and Fuchs 2020). *In vitro* reconstitution assays with purified proteins have shown that, when the Pol III core enzyme cannot catalyze nucleotide incorporation opposite damaged templates, it can be replaced by TLS DNAPs. These enzymes possess open and flexible active sites that accommodate bulky DNA lesions, permitting lesion bypass and replication continuation (Goodman and Woodgate 2013, Timinskas and Venclovas 2019)—albeit at the cost of clusters of mutations (collateral mutagenesis) (reviewed in Fujii and Fuchs 2020).

Live-cell studies have shown that TLS DNAPs do not spontaneously associate with the nucleoid, likely due to insufficient levels of damage-inducible TLS DNAPs to effectively compete with Pol III (Tuan et al. 2022). However, spontaneous mutagenesis *via* Pol IV or Pol V has been documented (Goodman and Woodgate 2013). When ssDNA regions persist, the SOS response is triggered, increasing the expression of TLS DNAPs (Pol II, Pol IV, and Pol V) (Sassanfar and Roberts 1990, Courcelle et al. 2001). These enzymes become enriched in the nucleoid, where 90% of Pol IV and 95% of Pol V foci localize distal to replisomes to facilitate lesion bypass *via* error-prone DTT subpathways (Fig. 1(h)) (Robinson et al. 2015, Thrall et al. 2017, 2022, Henrikus et al. 2018b). There is a functional division of labor between these TLS DNAPs: Pol IV contributes to uninduced mutagenesis, survival after MMS treatment and the error-prone bypass of MMS lesions, whereas Pol V is responsible for nearly all UV-induced mutagenesis and the error-prone bypass of MMS lesions, which is highly increased in a Δ*dinB* strain (Bjedov et al. 2007, Fuchs and Fujii 2013, Goodman and Woodgate 2013, Henrikus et al. 2018b).

RecA plays a regulatory role in this process by inhibiting Pol III* while activating Pol II, Pol IV, and Pol V facilitating DNAP exchange through interaction with the DnaN/β-sliding clamp *in vitro* (Indiani et al. 2013). Pol IV interacts with SSB bound to ssDNA and effectively competes with DnaQ/ε for binding to the DnaN/β-sliding clamp, a process facilitated by DnaXZ/γ (Kath et al. 2014, Thrall et al. 2022, Tuan et al. 2022). In contrast, Pol V, which initially has minimal TLS activity, becomes activated through interaction with RecA·ATP bound to the 3′-proximal end. Its UmuC subunit is sequestered at cellular membranes. In the presence of RecA·ATP nucleoprotein filaments, the UmuD homodimer (UmuD_2_) undergoes autocatalytic cleavage generating UmuD’_2_. This enables the assembly of the heterotrimeric UmuCD’_2_ complex, which is released into the cytosol but still exhibits limited TLS activity (Goodman 2002, Patel et al. 2010, Robinson et al. 2015). Full activation of the Pol V mutasome occurs only upon transfer of RecA·ATP bound to the 3′-proximal end (Patel et al. 2010). The resulting UmuCD′_2_-RecA·ATP–ssDNA complex (*a.k.a*. Pol V Mut) interacts with the DnaN/β-sliding clamp to bypass DNA lesions, and contributes to untargeted mutagenesis (Courcelle et al. 2005, Patel et al. 2010, Erdem et al. 2014, Kath et al. 2014). Thus, RecA may serve as a molecular switch, indirectly regulating the RS response by modulating the access of different TLS DNAPs to postreplicative lesion-containing gaps, adding an additional layer of RS control (McInerney and O’Donnell 2007).

Pol II possesses both DNAP and 3′→5′ exonuclease proofreading activities, functioning as an accessory DNAP capable of synthesizing across damaged DNA to some extent in an attempt to continue DNA replication (Rangarajan et al. 1999, Wang and Yang 2009, Henrikus et al. 2018a, Fujii and Fuchs 2020). Pol II interacts with DnaN/β-clamp *via* its CBM, competing with DnaQ/ε, to transiently bypass the template lesion, resulting in a slow-moving RF *in vitro* (Lopez de Saro et al. 2003, Indiani et al. 2009, Kath et al. 2016, Chang et al. 2019, 180). However, whether Pol II colocalizes with replisome markers in DNA-damaged cells remains to be determined.

### Fork remodeling and lesion bypass at stalled forks in *B. subtilis*

The *B. subtilis* replisome disassembles at leading- or lagging-strand template barriers, as indicated by the stoichiometries of the replicative helicase DnaC and the PolC HE (*i.e*. PolC or DnaX), showing that only one active replisome is observed in >40% of unperturbed cells (Mangiameli et al. 2017a). Replisome average residence time (*e.g*. PolC and DnaX) is on the scale of seconds in unperturbed cells but becomes significantly shorter upon blocking or inhibiting PolC or upon fork stalling at DNA damage sites (Liao et al. 2016, Hernández-Tamayo et al. 2019).

RecA, RecO, RecR, RecF, RarA, DisA, PolA, and PolY1 form foci that colocalize with replisome markers following DNA damage (Romero et al. 2019b, Simmons et al. 2007, Lenhart et al. 2014, Yeesin 2019). RecA assembled at stalled RFs may prevent unregulated degradation of the stalled RF, thereby preserving genome integrity. RecA physically interacts with and may contribute to recruiting several fork-remodeling factors, including the RecG branch migration translocase, the RuvAB–RecU resolvasome complex, and the RecD2 and RadA/Sms helicases. These homologous recombination proteins may promote lesion circumvention *via* error-free DDT subpathways (fork reversal and template switching) (Fig. 1(c) and (d)) (reviewed in Carrasco et al. 2024). In addition, RecA may recruit DisA, which physically interacts with RadA/Sms (Torres et al. 2019b, Gándara and Alonso 2015). DisA and RadA/Sms synergistically inhibit canonical RecA activities (ATP hydrolysis and DNA strand exchange) (Torres et al. 2019a, b). These findings support the idea that noncanonical activities of RecA act as local responders to a wide range of RS types and as global responders in a subset of them (Torres et al. 2019b). However, despite advances in understanding the dynamic protein choreography at stalled forks, several questions remain open.

Fork reversal is a protective fork-remodeling mechanism mediated by DNA translocases and/or DNA helicases (Fig. 1(c)), as RecG, RuvAB, and RecD2, or RadA/Sms, respectively (Cañas et al. 2014, Torres and Alonso 2021, Ramos et al. 2022, Hormeño et al. 2025). They may convert stalled RFs into reversed forks by coordinating the annealing of template strands, thereby extruding the newly synthesized nascent strands as a short branch, forming HJ-like structures. The extension of DNA synthesis on the nascent leading-strand using as template the intact complementary strand at the reversed RF bypasses the lesion, followed by fork restoration (Fig. 1(i) and (j)) (Carrasco et al. 2024). Live-cell studies have shown that RTCs lead to RF stalling and reversal across the conflict region in the absence of exogenous threats (Stoy et al. 2023), consistent with the observation that spontaneous RecA foci are present in ∼15% of wt cells growing in LB medium (Simmons et al. 2007). Following RF blockage by an engineered HO RTC, RF reversal at R-loops occurs in ∼12% of cells, and significantly increases in Δ*rnhC* cells (Fig. 1(c)) (Stoy et al. 2023). However, the frequency of fork reversal in response to exogenous stress remains unknown.

Translocases do not seem to perform redundant pathways, because a Δ*recG* mutation is synthetically lethal in the Δ*ruvAB* or Δ*recD2* backgrounds, depletion of RecD2 strongly reduced viability, >500-fold, in the Δ*ruvAB* or of Δ*recG* context, whereas *radA* is epistatic with *recG* or *ruvAB*, but not with *recD2* (Sanchez et al. 2005, Gándara et al. 2017, Raguse et al. 2017, Torres et al. 2017). Furthermore, the *disA* gene is epistatic with *recA, recG, radA*, or *ruvB* in response to RS (Gándara et al. 2017, Raguse et al. 2017). DisA, which binds branched intermediates, restrains RecG-mediated fork remodeling, RecG- or RuvAB-mediated fork restoration, and RuvAB–RecU-mediated resolution of the HJ structure *in vitro* (Gándara et al. 2021, Torres and Alonso 2021, Torres et al. 2023). The coordination of these proteins is complex and not fully understood.

Since only a fraction of stalled RFs is reversed, other fork remodeling pathways, such as template switching or strand transfer, should also contribute to overcoming RS (Torres et al. 2023, Carrasco et al. 2024). Template switching, which may involve homologous recombination factors including RecA, enables the nascent strand to anneal to its complementary nascent strand (Fig. 1(d)). Then, the latter serves as a template instead of the damaged parental strand, ensuring the correct sequence is copied from an undamaged template. A translocase may subsequently catalyze fork restoration, allowing the newly synthesized strand to return to its original position (Fig. 1(j)).

Lesions at stalled forks may be bypassed by error-prone DDT subpathways mediated by TLS DNAPs, that in *B. subtilis* are PolY1, PolY2, PolA, and DnaE (Fig. 1(e)). Approximately 30% of PolY1 and PolA molecules are spontaneously static and selectively enriched at or near replisome markers in unperturbed cells. Following DNA damage, the localization and dynamics of PolY1 change minimally; however, the proportion of static PolA molecules increases to ∼43% (Hinrichs and Graumann 2024, Marrin et al. 2024). Given that RecA assembles at stalled forks (Simmons et al. 2007), and that PolA interacts with RecA, PolY1, PolY2, and DnaN (Duigou et al. 2005, Lenhart et al. 2014, Carrasco et al. 2025), it is plausible that RecA and/or DnaN recruit TLS DNAPs to stalled forks. Notably, the static PolY1 population decreases ∼3-fold and does not colocalize with replisome markers when its interaction with the DnaN-sliding clamp is disrupted (as in PolY1-clamp-binding motif-mut2 mutant) (Marrin et al. 2024). Persistent RF stalling induces PolY2 expression, and to a lesser extent DnaE expression, in a LexA-dependent manner (Ohmori et al. 2001, Au et al. 2005). Live-cell fluorescence microscopy studies with the PolY2 enzyme have not yet been reported. Such studies have revealed that the essential DnaE TLS DNAP exhibits a very short dwell time, in contrast to PolC (Li et al. 2019), and dissociates from stalled forks in response to DNA damage (Li et al. 2019, Hernández-Tamayo et al. 2021). This is consistent with the minor contribution of DnaE in extending RNA primers before handing them off to PolC (Sanders et al. 2010, Seco and Ayora 2017). However, the role of DnaE, and its potential partners in mutagenic repair remain poorly understood.

Available genetic evidence suggests that TLS DNAPs function as bipartite enzymes—PolY1-PolA and PolY2-PolA—since: (i) deletion of the specific interacting domain (SID) with PolY1 or PolY2 in PolA results in mutation rates comparable to those of wt cells (Duigou et al. 2005, Carvajal-Garcia et al. 2023); (ii) spontaneous and damage-induced mutagenesis is inhibited in the Δ*polA* strain and is blocked in the Δ*polA* Δ*polY1* and Δ*polA* Δ*polY2* strains (Sung et al. 2003, Duigou et al. 2004, 2005, Raguse et al. 2017, Carrasco et al. 2025); and (iii) ternary complexes involving PolA, DnaN, and either PolY1 or PolY2 have been detected using yeast three-hybrids assays (Duigou et al. 2005). Additionally, PolA interacts with PolC, DnaE, and HolB *via* SID (Noirot-Gros et al. 2002, Duigou et al. 2005). Spontaneous or exogenous [UV- (or 4NQO) or MMS-induced] damage halts and disassembles the high fidelity replicative PolC (Mangiameli et al. 2017a). If error-free DDT subpathways become overwhelmed, PolY1 or PolY2, in concert with PolA, may bypass the lesion at or near the RF. Within these bipartite DNAPs, PolY1 and PolY2 catalyse stable, though often erroneous, nucleotide incorporation opposite damaged templates, and PolA extends the nascent strand and fixes the error by protecting it from nucleases (Duigou et al. 2005). Subsequently, PolA hands-off the DNA to the replicative DNAP, which can sense and efficiently proofread the misincorporated nucleotide (Fig. 1(e) and (k)) (Sanders et al. 2010). It remains unclear whether PolY1 or PolY2 can independently compete with transiently paused PolC to bypass lesions, and how such error-prone bipartite TLS DNAP (PolY1–PolA and PolY2–PolA) complexes are regulated. Interestingly, the spontaneous mutation rate in Δ*polY1* or Δ*polY2* strains is comparable to that of untreated wt cells. Upon UV- (or 4NQO-) or MMS-induced damage, the mutation rate is similar or only modestly reduced compared to wt cells, suggesting that PolY1 and PolY2 exhibit a certain functional redundancy. Both spontaneous and damage-induced mutagenesis are severely impaired in the Δ*polY1* Δ*polY2* strain (Duigou et al. 2005, Raguse et al. 2017). Most UV-induced mutagenesis by PolY2 depends on PolA, although a minor fraction (<10%) of UV and spontaneous PolY2-induced mutagenesis occurs independently of PolA (Duigou et al. 2005, Murray et al. 2017). The PolY1–PolA or PolY2–PolA complexes, together with RecA, require the DnaN sliding clamp for spontaneous mutagenesis; however, DnaN is dispensable for UV-induced mutagenesis by PolY2 (Duigou et al. 2004, Murray et al. 2017).

The choice of DDT subpathways at stalled RFs should be coordinated with replication restart. In *in vitro* reconstitution replication assays using a stalled fork substrate, RarA or RecA, together with RecO and SsbA, restrains PriA-dependent replication restart without affecting replication elongation. This inhibition is counteracted by RecD2 or PcrA (Million-Weaver et al. 2015a, Vlasic et al. 2014, Carrasco et al. 2022, Ramos et al. 2022). It seems that RecA contributes to the loading of preprimosome proteins necessary for replication reinitiation at stalled forks, at least in response to RTCs (Million-Weaver et al. 2015a). PriA is the key factor for replication restart in *B. subtilis*. In fact, depletion of PriA for roughly one doubling time prevents replication restart after spontaneous fork disassembly in ∼90% of unperturbed cells (Mangiameli et al. 2017a). PriA facilitates replication restart by recognizing and binding branched DNA intermediates, including: (i) 5′-forks, lacking a nascent leading strand, that arise when RFs stall al leading-strand template lesions; (ii) 3′-forks, lacking a nascent lagging strand, that arise when RFs stall at lagging-strand template lesions; and (iii) replicated forks, with both nascent strands, that arise at protein roadblocks or RTCs. PriA-dependent reloading of a single DnaC–DnaI complex at stalled forks uses a similar set of preprimosomal proteins as those involved in DnaA-dependent replication initiation at *oriC2*. PriA bound to a branched intermediate interacts with and promotes the loading of DnaD, which in turn recruits DnaB, forming a preprimosome (PriA–DnaD–DnaB) complex. In coordination with the DnaI chaperone, this complex loads the replicative helicase DnaC (Masai et al. 1999, Ishigo-Oka et al. 2001, Marsin et al. 2001, Polard et al. 2002, Bruand et al. 2005). Subsequently, DnaC recruits the remaining components of the replisome *via* protein–protein interactions (Murray et al. 2017).

Does *B. subtilis* tolerate DNA damage through repriming and subsequent postreplicative gap filling? There is evidence of stalled replisome disassembly, and recruitment of RecA, its accessory proteins, and the error-prone DNAPs PolY1 and PolA, at or near replisome markers in response to RS (Romero et al. 2019b, Simmons et al. 2007, Lenhart et al. 2014, Yeesin 2019, Hinrichs and Graumann 2024, Marrin et al. 2024). Furthermore, putative RecA-independent DDT mechanisms, like the RarA- and DnaK-dependent pathways described in *E. coli* (Cox et al. 2023), may not be functional in *B. subtilis*. In fact, RarA assembles at stalled RFs and contributes to RecA filament growth, and has an undefined role in modulating error-prone DDT subpathways (Romero et al. 2019a, b, 2020). Meanwhile, DnaK is typically localized near the cell membrane or poles rather than at the nucleoid (Meile et al. 2006), and primarily functions in protein quality control and the mitigation of proteotoxic stress (Matavacas et al. 2022). Lesion skipping is likely a minor mechanism in *B. subtilis* wt cells for overcoming RS.

## Conclusions and future perspectives

**Figure 3.**
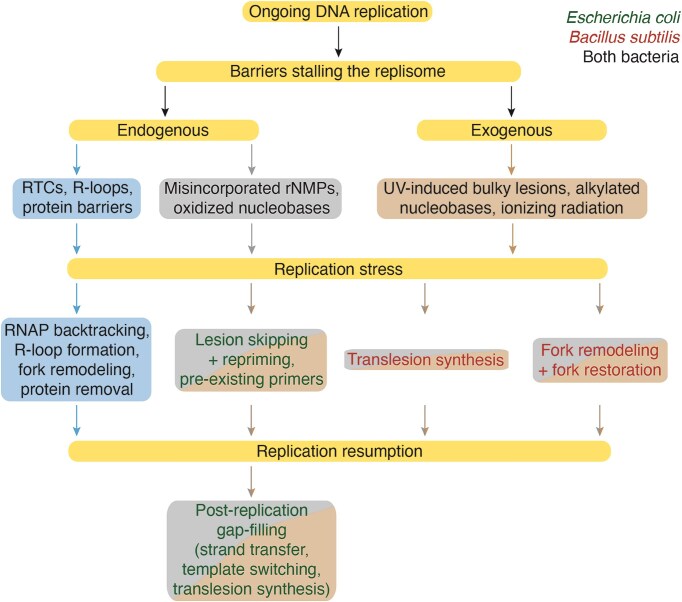
Summary of the responses to RS in *E. coli* and *B. subtilis*. Mechanisms specific to *E. coli* are shown in green, those specific to *B. subtilis* in red, and shared aspects in black. The arrow and box colors indicate the directional flow of the corresponding processes.

Genetic approaches, live-cell imaging by time-lapse microscopy, and biochemical studies have substantially contributed to our understanding of the responses to RS in both *E. coli* and *B. subtilis* cells (Fig. 3). In this review, we have compiled these data to describe the dynamic spatio-temporal order of protein assembly specifically at stalled RFs in the presence of a vast excess of DNA without such obstacles, and the mechanisms used to circumvent or bypass the barrier.

Multiple lines of evidence emphasize that the central mechanisms by which bacteria cope with RS are generally conserved among phylogenetically distant bacteria, although it remains unclear whether this conservation results from parallel or convergent evolution. However, these distantly related bacteria have evolved specific mechanisms to sense and recognize DNA impediments, enabling them to adapt to their specific challenges in response to endogenous and exogenous threats. Consequently, findings from one bacterium cannot be directly extrapolated to the other. Understanding these similarities and differences will provide a comprehensive overview of how stalled RFs are stabilized and the molecular mechanisms governing the commitment to different DDT subpathways.

Both model bacteria use a variety of mechanisms to circumvent or bypass offending insults, recover RFs, maintain fork stability, and facilitate the restart of stalled RFs. Endogenous threats to RF progression mostly trigger local responses, whereas exogenous threats induce both local and global responses through LexA-dependent and less well-characterized LexA-independent mechanisms. Moreover, the mechanisms of fork reactivation differ significantly between the two model bacteria. In *E. coli*, lesion skipping upon *de novo* repriming is the predominant strategy, where recombination proteins mainly assemble at the lesion-containing gap distal to the replisomes. This postreplication repair pathways then converts these gaps into duplex DNA through both RecA-dependent and RecA-independent mechanisms, followed by specialized excision repair to remove the lesion. If lesion skipping fails, such as when the replisome collides with the transcription machinery, the RF is remodeled and replication restarts in a RecA-independent manner. In contrast, the *B. subtilis* replisome disassembles upon encountering a blockage, with RecA protecting the stalled fork. Error-free and error-prone DNA DDT subpathways act directly at the stalled fork to overcome the RS and facilitate replication restart, followed by specialized excision repair to remove the lesion. Notably, *B. subtilis* and mammalian cells share a propensity for frequent fork reversal, involving numerous specialized proteins that contribute to the formation and stabilization of reversed forks.

Despite significant progress, many questions remain largely unanswered and should be the subject of future studies. For instance, it will be of significant interest to define the potential noncanonical activities of recombination proteins, such as RecA, at stalled forks in both bacteria and to understand how they are recruited to specific locations at replisomes or distal from them. Moreover, it remains unknown why *B. subtilis* favors fork remodeling at stalled forks and reconstitutes the RF, rather than simply skipping the barrier and subsequently removing it later through different postreplicative mechanisms, as *E. coli* primarily does.
